# Multivariate elucidation of soil-microbial-physiological interactions under bio-organic nutrient modules in kiwifruit (*Actinidia deliciosa* A. Chev.)

**DOI:** 10.1186/s12870-026-08259-6

**Published:** 2026-04-02

**Authors:** Manish Nagu, Vishal Singh Rana, Dharam Paul Sharma, Rajesh Kaushal, Anjali Chauhan, Himanshu Mehta, Shivanshu Garg, Saurabh Gangola, Faheema Khan, Shifa Khan, Kahkashan Perveen

**Affiliations:** 1 Department of Fruit Science, Dr. YS Parmar University of Horticulture and Forestry, Nauni, 173230 Himachal Pradesh India; 2 Department of Soil Science and Water Management, Dr. YS Parmar University of Horticulture and Forestry, Nauni, 173230 Himachal Pradesh India; 3 School of Agriculture and Technology, Maya Devi University, Dehradun, 248011 Uttarakhand India; 4 Department of Biochemistry, Lovely Professional University, Phagwara, 144411 Punjab India; 5 Department of Microbiology, Graphic Era University, Dehradun, 248002 Uttarakhand India; 6 Department of Botany and Microbiology, College of Science, King Saud University, P.O. Box 22452, Riyadh, 11451 Saudi Arabia; 7 The Kania School of Management, The University of Scranton, Brennan Hall 400, 320 Madison Avenue, Scranton, PA 18510 USA

**Keywords:** Organic, *Jeevamrit*, Multivariate analysis, Sustainability

## Abstract

**Background:**

Organic formulations are emerging as sustainable and eco-friendly solutions for enhancing crop production and promoting long-term agro-ecosystem resilience. The present investigation was carried out on kiwifruit cv. ‘Allison’ under mid-hill conditions of Himachal Pradesh. The study aimed to assess the influence of integrated nutrient management modules comprising organic manures, biofertilizers (UHF-Jeevanu Khad) and *Jeevamrit*-based formulations on plant physiological traits, soil physico-chemical and chemical properties, and microbial activity.

**Results:**

A field experiment, laid out in a randomized block design (RBD) with seven treatments and three replications, revealed significant improvements in physiological parameters, specifically, chlorophyll content, photosynthetic rate, stomatal conductance and transpiration rate, which collectively enhanced overall plant metabolic efficiency. Among the treatments, the combination of 75% recommended dose of fertilizers (RDF) and 25% UHF-Jeevanu Khad significantly enhanced soil macronutrients, namely N, P and K by 17.75, 20.58 and 19.78%, respectively, over their sole application, along with notable improvements in micronutrients (Fe, Zn, Mn, Cu), indicating improved nutrient dynamics. Microbial populations, including bacteria, fungi and actinomycetes, increased substantially under organic inputs, indicating enhanced microbial activity in the rhizosphere, which may improve root-microbe interactions and nutrient cycling. Multivariate statistical analyses including principal component analysis (PCA) and heatmap cluster revealed strong associations among microbial, nutrient and physiological traits, with the treatment comprising 75% RDF combined with 25% UHF-Jeevanu Khad, and the treatment involving RDF at 800:600:800 g N:P:K per vine forming distinct, high-loading clusters. Correlation analysis showed significant positive relationships between available nitrogen and photosynthetic rate (*r* = 0.983), stomatal conductance (*r* = 0.964) and chlorophyll content (*r* = 0.958), indicating close linkage between nutrient availability and plant physiological performance. Additionally, multiple regression analysis (R^2^ = 0.9938) identified available nitrogen and photosynthetic traits as dominant predictors of yield.

**Conclusion:**

The integration of organic manures, biofertilizers and *Jeevamrit*-based formulations with recommended fertilizers enhanced soil fertility, microbial activity and plant physiological performance, thus supporting sustainable kiwifruit production in mid-hill regions. These findings underline the importance of combining organic and microbial inputs with conventional practice to improve nutrient cycling, plant health and productivity.

**Supplementary Information:**

The online version contains supplementary material available at 10.1186/s12870-026-08259-6.

## Introduction

Rapid population growth has outpaced food supply, worsening undernutrition and intensifying pressure on scarce arable land and natural resources essential for crop production [1]. Furthermore, reliance on synthetic fertilizers has resulted in the depletion of soil organic matter, nutrient imbalances, degradation of soil structure and a decline in beneficial microorganisms essential for nutrient cycling [2]. Additionally, continuous monocropping, inadequate use of organic amendments, declining soil biodiversity, and inefficient irrigation practices have further exacerbated soil degradation and reduced long-term sustainability of orchard ecosystems [3]. Given India’s significant fertilizer imports and environmental concerns, there is an urgent need to adopt sustainable nutrient management approaches [4]. While the Green Revolution improved national food security through increased productivity, its heavy focus on chemical inputs led to nutrient imbalances, micronutrient deficiencies and gradual declines in soil health, posing long-term sustainability concerns [5]. Recent studies in perennial orchard ecosystems demonstrate that fertilizer-intensive management leads to substantial ecological disruptions, for instance, kiwifruit orchards under conventional fertilization experienced over 37% higher CO_2_ emissions, five-fold greater losses of dissolved inorganic nitrogen and nearly 80% reduction in earthworm populations compared to organic or integrated systems [6]. Greater emphasis on managing rhizosphere microbial activity and biomass is crucial for ensuring agricultural sustainability and profitability [7].

To address these challenges, Integrated Nutrient Management (INM**)**, which combines organic amendments, biofertilizers, and reduced chemical inputs, has been recognized globally for its capacity to restore soil fertility while sustaining or improving yields [8]. Organic materials such as farmyard manure (FYM), vermicompost, poultry manure and Indian traditional organic formulations like *Jeevamrit* and *Ghan Jeevamrit* supply vital macro- and micro-nutrients and stimulating microbial biomass and enzymatic activities [9–11]. Studies have demonstrated that the application of bio-organic fertilizers significantly improves soil biological properties by enhancing soil enzyme activities and microbial diversity [12]. Organic manures such as FYM and vermicompost are rich in humus and essential nutrients and naturally harbor beneficial microorganisms including *Azotobacter*, *Azospirillum* and phosphate-solubilizing bacteria [13]. Vermicompost ensures a slow and steady release of essential macronutrients, enhances soil physical properties, and facilitates efficient nutrient uptake, thereby promoting better crop growth and yield [14]. *Jeevamrit*, a traditional microbial bio-formulation prepared using cow dung, cow urine, jaggery, legume flour and soil, has been scientifically shown to enhance soil fertility and plant productivity by increasing microbial biomass, stimulating key microbial genera such as *Bacillus* and *Pseudomonas*, and elevating soil enzyme activities involved in nutrient cycling [15–18]. In addition, the application of *Jeevamrit* has demonstrated the potential to mitigate abiotic stresses, such as salinity and drought, while improving plant growth across various cropping systems [19]. Poultry manure not only reduces reliance on synthetic fertilizers and lowers pollution but also improves soil structure, fertility, and organic carbon content, contributing to better soil health and reduced greenhouse gas emissions [20]. Organic fertilizers supply nutrients while improving soil structure and microbial habitats thereby enhancing the soil physical, chemical and biological properties [21, 22]. Meta-analysis confirmed that organic amendments consistently improve soil microbial functionality, nutrient cycling and overall productivity across diverse agroecosystems [23].

Kiwifruit (*Actinidia deliciosa* Chev.), an economically significant fruit crop, is valued for its rich nutritional composition and health-promoting properties, including high vitamin C content and essential minerals [24, 25]. In India, kiwifruit cultivation is concentrated in the mid-hills of Arunachal Pradesh, Uttarakhand, Sikkim, Mizoram, Nagaland, Jammu & Kashmir and Himachal Pradesh, where interest is increasing as farmers diversify due to its high yield potential and market value [26].

However, productivity in Indian kiwifruit orchards is increasingly constrained by soil acidification, declining organic carbon and low microbial activity, consequences of excessive and prolonged use of synthetic fertilizer. In the mid-hill agro-ecologies of Himachal Pradesh, where kiwifruit cultivation is expanding, integrating locally available organic nutrient sources offers significant potential to improve soil fertility and sustain yields. Although individual organic amendments such as FYM, vermicompost, poultry manure, *Jeevamrit* and *Ghan Jeevamrit* have shown promise in improving soil and plant parameters, systematic studies evaluating their combined use as integrated organic nutrient modules in kiwifruit-based systems are lacking. Notably, existing research has largely been conducted either on single organic inputs or within unrelated horticultural systems, leaving a critical gap in understanding the synergistic effects and region-specific suitability of multi-component organic nutrient strategies under the unique climatic and topographical conditions of the Himachal mid-hills.

Given that soil microbial activity is central to nutrient cycling and orchard health, this study aimed to comprehensively assess how different organic nutrient modules influence soil properties and microbial populations under mid-hill conditions of Himachal Pradesh. In addition, the study evaluated their effects on kiwifruit physiological and yield performance. To better understand the inter-relationships among these variables, multivariate analysis including correlation, regression and PCA was conducted to identify key soil and microbial indicators associated with improved kiwifruit productivity. These analyses together provide region-specific insights to inform sustainable nutrient management and orchard management strategies for kiwifruit cultivation in Himachal Pradesh’s mid-hills.

## Materials and methods

### Study area

The experiment was conducted at the Experimental Block of the Department of Fruit Science, Dr YS Parmar University of Horticulture and Forestry, Nauni, Solan, Himachal Pradesh. The site is located at 30º 50ʹ N latitude and 77º 11ʹ 30ʺ E longitude, at an elevation of 1260 m above mean sea level. It falls within the sub-temperate, sub-humid mid-hill agro-climatic zone of Himachal Pradesh (Zone-II). The area receives an average annual rainfall of 1000–1300 mm, primarily during the monsoon months of July to September. The climate is characterized by moderately warm summers during May and June and cold winters from December to January.

### Edaphic conditions

The experimental plot soil is classified as brown hill soils [27], Typic Haplustepts (USDA) and Cambisols (FAO-UNESCO). The soil was sandy loam in texture, comprising sand, silt and clay at 55.24, 29.00 and 20.76%, respectively, with medium to high organic matter and a neutral to slightly acidic pH (6.76). It had an electrical conductivity (EC) of 0.20 dS m^− 1^, organic carbon content of 1.36% and moderate fertility, with alkaline KMnO_4_ extractable N at 329.87 kg ha^− 1^, available NaHCO_3_-extractable P at 66.55 kg ha^− 1^, and available NH_4_OAc-K at 516.00 kg ha^− 1^. Diethylene triamine pentaacetic acid (DTPA) extractable micronutrients cations viz., Fe, Cu, Zn and Mn were 1.64, 34.1, 42.6 and 1.49 mg kg^− 1^. The relatively higher DTPA-extractable micronutrient concentrations reflect enhanced plant-available fractions under long-term organic manure-amended orchard soils. The experimental soil had an initial viable microbial population of bacteria (4.1 × 10^4^ colony-forming units (cfu) g^− 1^), fungi (6.3 × 10^3^ cfu g^− 1^) and actinomycetes (9.5 × 10^4^ cfu g^− 1^).

### Experimental design

The experiment was conducted on 10-year-old uniformly vigorous Allison kiwifruit vines, a cultivar preferred in India for its early flowering, early maturity, prolific fruiting and superior yield potential. Vines were spaced at 4.0 m x 6.0 m with a pollinizer (male Allison) at a 1:9 ratio and trained on the T-bar system. Uniform cultural practices were followed throughout the study. The experiment was laid out in an RBD, consisting of seven treatments replicated three times. The treatments included: T_1_ - Vermicompost (50%) + Poultry manure (50%), T_2_ - Vermicompost (40%) + Poultry manure (60%), T_3_ - Vermicompost (60%) + Poultry manure (40%), T_4_ - *Jeevamrit* @ 30 L per vine + *Ghan Jeevamrit* @ 3 kg per vine, T_5_ - UHF-Jeevanu Khad (a *Bacillus*-based biofertilizer developed at Dr. Yashwant Singh Parmar University of Horticulture and Forestry, Nauni) @ 5 L per vine, T_6_ − 75% RDF + 25% UHF-Jeevanu Khad and T_7_ - RDF @ 800:600:800 g N:P:K per vine. In treatments T_1_-T_3_, vermicompost and poultry manure were applied based on the proportion of nitrogen they supplied relative to the Recommended Dose of Nitrogen (RDN).

The full recommended dose of FYM, single super phosphate (SSP) and muriate of potash (MOP) was applied during the first fortnight of January. Vermicompost and poultry manure were applied in three equal splits during the last week of January, February and April. *Jeevamrit* and *Ghan Jeevamrit* were also applied in three splits at the end of January, February, and April. The full dose of UHF-Jeevanu Khad (*Bacillus licheniformis* strain B6 @ 10^8^ cfu ml^− 1^) was applied during the last week of January.

### Bio-organic formulations

Vermicompost and UHF-Jeevanu Khad (a biofertilizer composed of *B. licheniformis* strain B6 (10^8^ cfu ml^− 1^), possessing plant growth-promoting traits such as phosphate solubilization, Indole-3-acetic acid (IAA) and siderophore production, and antifungal traits) was procured from the Department of Soil Science and Water Management, Dr. Y. S. Parmar University of Horticulture and Forestry, Nauni, Solan, Himachal Pradesh, India (30° 51ʹ 24.45ʺ N, 77° 10ʹ 0.62ʺ E) and poultry manure was sourced from a certified local supplier in Solan district, Himachal Pradesh (30° 55ʹ 4.89 ʺ N, 77° 6ʹ 26.20 ʺ E. *Jeevamrit* and *Ghan Jeevamrit* were prepared following the procedure described by [28]. *Jeevamrit* was made by taking 200 L of water in a barrel, adding 10 kg fresh local cow dung, 10 L cow urine, 2 kg jaggery, 1 kg pulse flour and 1 kg soil from the bund of the farm. The mixture was stirred in clockwise direction for 5–10 min twice daily and fermented for 3–5 days before use. *Ghan Jeevamrit* was prepared by mixing 100 kg local cow dung with 2 kg jaggery, 2 kg pulse flour, a handful of soil from the bund and a small amount of cow urine. The mixture was shade-dried, powdered, and stored in plastic drums until application.

### Vegetative attributes

Vegetative attributes were recorded during the active growth period [29]. Leaf area was measured in June using a LI-COR 3100 m on ten fully expanded leaves per vine (cm^2^ leaf^− 1^). Vine girth was measured at 30 cm above ground before and after the season using a measuring tape (cm). Cane diameter was recorded after pruning using a Digital Vernier Calliper (mm shoot^− 1^).

### Physiological traits

Chlorophyll content was estimated using the method of Hiscox and Israelstam [30]. Ten healthy leaves from each tree were collected in the third week of June [31] and stored below 0 °C. Ten milligrams of leaf tissue were extracted in 10 mL DMSO at 65 °C for 3 h. Absorbance was measured at 645 and 663 nm using Nukes UV-VIS spectrophotometer and total chlorophyll (mg g^-1^ FW) was calculated using [32] equation, total chlorophyll = 20.2 × A_645_ + 8.02 × A_663_. Photosynthetic rate, stomatal conductance, and transpiration rate were recorded from 10:00–12:00 AM in June using the LI-6400XT Portable Photosynthesis System (LI-COR Inc., USA). Results were expressed in µmol CO_2_ m^-2^ s^-1^, mol H_2_O m^-2^ s^-1^ and mmol H_2_O m^-2^ s^-1^, respectively.

### Yield characteristics

Fruit yield was recorded as total weight per vine (kg vine^− 1^). Harvested fruits were graded by weight into Grade A (> 80 g), Grade B (60–80 g) and Grade C (< 60 g), and grade-wise yields were expressed in kg vine^− 1^. Yield efficiency was calculated as yield per vine divided by trunk cross-sectional area (kg cm^− 2^) following Westwood [33]. Productivity was computed as yield per vine multiplied by vine density (374 vines ha^− 1^).

### Soil properties

Soil pH and EC were measured in a 1:2.5 soil-to-water suspension using a Eutech pH 700 m digital pH meter and EC 2700 conductivity meter [34]. Soil organic carbon was estimated following the Walkley and Black titration method [34]. The alkaline KMnO_4_ distillation method was used for available nitrogen estimation [35]. Available phosphorus quantification was done at 660 nm on a Nukes UV-VIS spectrophotometer after extraction with Olsen’s sodium bicarbonate solution [36]. Available potassium analysis was carried out using flame photometer after extraction with 1 N ammonium acetate [37]. Micronutrients (Fe, Zn, Cu, Mn) were analyzed on an Analyst 400 atomic absorption spectrophotometer after extraction with DTPA solution [38].

### Microbial assay

The cultivable microbial populations, expressed as cfu, were assessed using the standard plate count method [39]. Serial dilution was employed, with different culture media used to isolate specific microbial groups: nutrient agar for bacteria [40], potato dextrose agar for fungi and Kenknight and Munaier’s medium for actinobacteria. One gram of sieved (2 mm) soil was added to 9 ml of sterile water and shaken for 15–20 min to obtain a 10^− 1^ dilution. Serial dilutions ranging from 10^− 2^ to 10^− 7^ were prepared. From each dilution, 1 ml aliquots were spread onto cooled and solidified media in Petri plates, which were gently rotated for uniform distribution. Plates were incubated at 28 °C for 3–5 days [1], a temperature suitable for mesophilic soil microbes and aligned with the standard practice of incubating fungi and actinomycetes, while also allowing acceptable growth of soil bacteria under uniform conditions. Microbial populations were recorded as colony-forming units per gram (cfu g^− 1^) of soil.

### Statistical analysis

The data generated from the study were compiled, tabulated, and statistically analyzed using an RBD. The significance of differences among treatments was tested at the 5% probability level as per [41]. One-way ANOVA was performed using SPSS software to evaluate treatment effects, and post-hoc comparisons (*p* ≤ 0.05) were conducted using Duncan’s Multiple Range Test (DMRT) in R software (version 4.5.1).

To gain deeper insights into trait interactions, multivariate statistical analyses were employed. Correlation analysis was used to assess the relationships among soil, microbial and physiological traits. Multiple linear regression was applied to identify key predictors of fruit yield with yield as the dependent variable. Hierarchical cluster analysis grouped treatments and traits based on similarity patterns, while PCA was conducted to extract major components explaining trait variability and to visualize their association with yield. All multivariate analyses were performed using R software (version 4.5.1).

## Results and discussion

### Photosynthetic performance traits

**Fig. 1:**
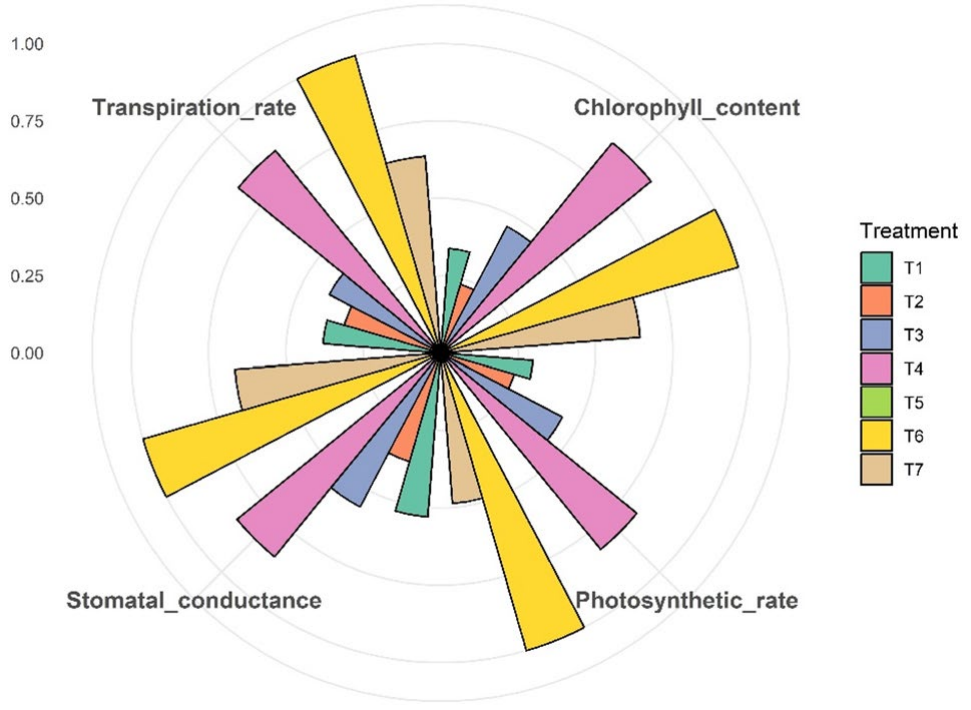
Radial bar plot of photosynthetic traits across organic nutrient modules in kiwifruit. *T₁, Vermicompost (50%) + Poultry manure (50%); T₂, Vermicompost (40%) + Poultry manure (60%); T₃, Vermicompost (60%) + Poultry manure (40%); T₄, Jeevamrit @ 30 L per vine + Ghan jeevamrit @ 3 kg per vine; T₅, UHF- Jeevanu Khad @ 5 L per vine; T₆, RDF (75%) + UHF- Jeevanu Khad (25%); T₇, RDF (800:600:800 g vine ⁻ ¹)*

Marked differences in photosynthetic performance traits were evident among the treatments, clearly reflecting the underlying physiological variation (Fig. 1). Vines under Treatment T_6_, which combined RDF (75%) with UHF-Jeevanu Khad (25%), consistently outperformed all other treatments, recording the highest chlorophyll content (3.16 mg g^-1^ FW However, vines under T_4_, comprising the natural farming-based formulations *Jeevamrit* and *Ghan Jeevamrit*, closely followed with a chlorophyll content of 3.08 mg g^-1^ FW, underscoring the potential of these low-input, eco-friendly bio-formulations as a promising alternative to conventional fertilizer-based systems. T_7_ (2.93 mg g^-1^ FW) and T_3_ (2.81 mg g^-1^ FW) also fared relatively well, whereas T_5_ lagged significantly with the lowest chlorophyll value (2.51 mg g^-1^ FW). T_6_ also exhibited the highest stomatal conductance (0.17 mol H_2_O m^-2^ s^-1^) and transpiration rate (4.93 mmol H_2_O m^-2^ s^-1^), closely followed by T4, which recorded 0.16 and 4.68 mmol H_2_O m^-2^ s^-1^, respectively. A similar trend was observed for photosynthetic rate, with T_6_ peaking at 14.36 µmol CO_2_ m^-2^ s^-1^ and T_4_ showing a comparable value of 13.46 µmol CO_2_ m^-2^ s^-1^. Conventional RDF under T_7_ showed moderate physiological efficiency, while T_5_ consistently recorded the lowest values across all traits.

These physiological advantages under 75% RDF combined with 25% UHF-Jeevanu Khad, followed by *Jeevamrit* @ 30 L per vine along with *Ghan Jeevamrit* @ 3 kg per vine, may be attributed to improved nutrient dynamics and enhanced physiological functioning. Increased leaf area under these treatments likely supported greater light interception, resulting in higher photosynthetic activity. This is further reinforced by the adequate supply of nitrogen and magnesium, key elements for chlorophyll formation and gas exchange efficiency [42]. Bio-organic formulations such as *Jeevamrit* and *Ghan Jeevamrit* are known to enhance soil moisture retention, organic carbon content and nutrient availability, which collectively improve water and nutrient uptake, thereby promoting higher stomatal conductance and transpiration rates [43]. Additionally, the inclusion of *Bacillus*-based bioinoculants in UHF-Jeevanu Khad possibly facilitated nutrient solubilization and uptake, contributing to the higher chlorophyll content and photosynthetic rate seen in T_6_. These findings are consistent with previous studies in banana and strawberry, where organic amendments improved nutrient uptake and gas exchange parameters [44, 45]. In kiwifruit, Liu et al. [46] demonstrated that combining no-tillage with organic fertilization enhanced microbial activity and soil properties, thereby improving vine physiology. Similarly, Raiesi et al. [47] observed increased chlorophyll concentration and photosynthetic efficiency with vermicompost application compared to chemical fertilizers. Supporting this trend, Mustafa et al. [48] reported that vermicompost combined with algae extract in mango resulted in higher leaf chlorophyll, stomatal conductance and transpiration, aligning closely with the physiological improvements detected under T_6_ in the current study.

### Yield attributes

**Fig. 2:**
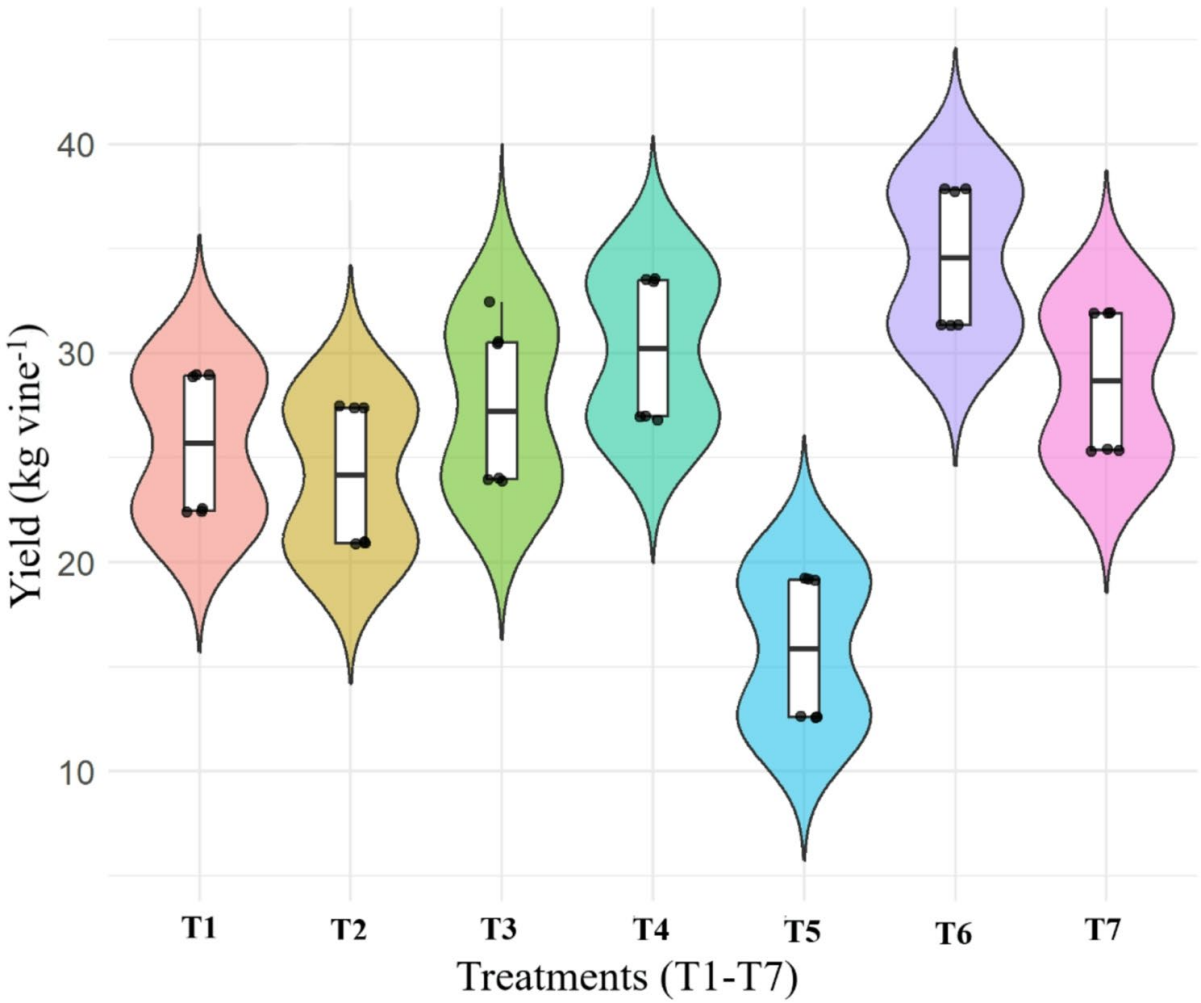
Violin plot showing treatment-wise distribution of total kiwifruit yield (kg vine^− 1^). *T₁, Vermicompost (50%) + Poultry manure (50%); T₂, Vermicompost (40%) + Poultry manure (60%); T₃, Vermicompost (60%) + Poultry manure (40%); T₄, Jeevamrit @ 30 L per vine + Ghan jeevamrit @ 3 kg per vine; T₅, UHF- Jeevanu Khad @ 5 L per vine; T₆, RDF (75%) + UHF- Jeevanu Khad (25%); T₇, RDF (800:600:800 g vine⁻¹)*

Among all Treatment T_6_ [RDF (75%) + UHF-Jeevanu Khad (25%)] exhibited the highest and most consistent yield, as indicated by its narrow interquartile range and higher median value (Fig. 2). In contrast, T_5_ (UHF-Jeevanu Khad @ 5 L vine^− 1^) showed the lowest yield with wider variability. The treatments comprising vermicompost (60%) + poultry manure (40%), *Jeevamrit* at 30 L per vine combined with *Ghan Jeevamrit* at 3 kg per vine and the application of RDF at 800:600:800 g N:P:K per vine also demonstrated relatively better performance compared to the treatments with vermicompost (50%) + poultry manure (50%) and vermicompost (40%) + poultry manure (60%). The violin plot clearly highlights the yield-enhancing effect of integrated nutrient application under T_6_ over sole organic or conventional practices.

**Fig. 3:**
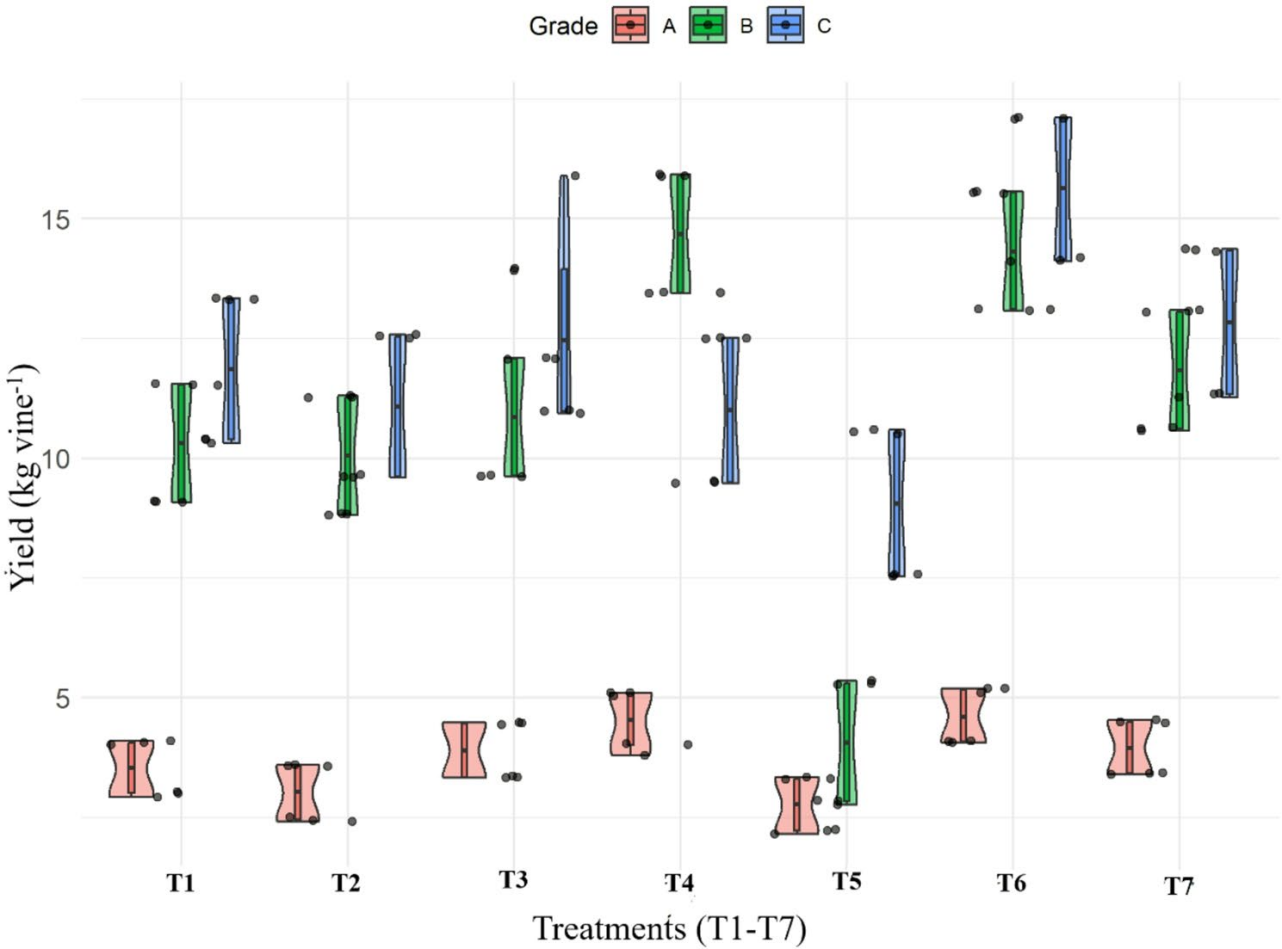
Grade-wise distribution of fruit yield under different treatments. *T₁, Vermicompost (50%) + Poultry manure (50%); T₂, Vermicompost (40%) + Poultry manure (60%); T₃, Vermicompost (60%) + Poultry manure (40%); T₄, Jeevamrit @ 30 L per vine + Ghan jeevamrit @ 3 kg per vine; T₅, UHF- Jeevanu Khad @ 5 L per vine; T₆, RDF (75%) + UHF- Jeevanu Khad (25%); T₇, RDF (800:600:800 g vine⁻¹)*

The data presented in Fig. 3 illustrate the differential influence of various organic nutrient regimes on the yield of grade-wise fruit in kiwifruit cv. Allison. A significant variation was observed in the production of Grade A fruits across treatments. The highest-Grade A yield (4.63 kg vine^− 1^) was obtained under T_6_ [RDF (75%) + UHF-Jeevanu Khad (25%)], followed closely by T_4_ (4.52 kg vine^− 1^), which involved *Jeevamrit* and *Ghan Jeevamrit*. These treatments outperformed others, suggesting that integration of organic inputs with either RDF or fermented bio-inoculants positively influenced the production of large-sized fruits. On the other hand, T_5_ (UHF-Jeevanu Khad @ 5 L vine^− 1^) recorded the lowest Grade A yield (2.76 kg vine^− 1^), indicating its limited effectiveness when applied alone. For Grade B fruits, treatment T_4_ led to the highest yield (14.68 kg vine^− 1^), significantly higher than the moderate yields recorded in T_1_ (11.38 kg vine^− 1^), T_3_ (11.97 kg vine^− 1^) and T_7_ (11.52 kg vine^− 1^). T_6_, although highly effective for Grade A and C fruits, recorded a slightly lower B-grade yield (10.74 kg vine^− 1^), suggesting a shift in fruit size distribution towards the extremes. In contrast, T_5_ again performed the least. Interestingly, the yield of Grade C fruits showed a reversed pattern in some treatments. T_6_ recorded the maximum yield (15.62 kg vine^− 1^), followed by T_7_ (13.62 kg vine^− 1^) and T_3_ (13.49 kg vine^− 1^). In contrast, lower Grade C yields were noted under T_2_ (10.17 kg vine^− 1^) and T_5_ (9.06 kg vine^− 1^).

The integration of the recommended fertilizer dose with UHF-Jeevanu Khad, rich in *Bacillus* strains, substantially improved kiwifruit yield, grade-wise fruit distribution and yield efficiency. This effect can be attributed to a balanced macro and micronutrient supply coupled with the phytohormone-producing potential of biofertilizers, which support enhanced fruit filling, metabolic activity and synthesis of key biomolecules like carbohydrates and proteins [49].

*Bacillus* spp. enhance yield via P solubilization and phytohormone production, thereby increasing sink strength and fruit enlargement. *Azotobacter* spp. are free-living N fixers that also synthesize growth regulators, improving assimilate supply to reproductive sinks and increasing fruit size and weight [50]. In addition, organic manures and biofertilizers enhance soil aggregation, moisture retention and nutrient bioavailability, which collectively elevate photosynthetic translocation and metabolic efficiency, critical for achieving higher fruit yields [51].

Similar findings in kiwifruit systems confirm the positive role of integrated nutrient management in promoting yield and quality under varied agroecological conditions [52]. The use of *Bacillus*-based plant growth-promoting rhizobacteria (PGPR) has also demonstrated improvements in nutrient uptake, root health and stress resilience in banana [53] and strawberry [45]. In strawberry, Bhat et al. [54] reported a marked increase in fruit yield and quality with *Bacillus*-based bioinoculants, reaffirming the multifunctional role of these microbes in reproductive enhancement. Likewise, Okba et al. [55] observed that the substitution of 25–50% of NPK with compost and microbial consortia (e.g., *Azotobacter*, *Azospirillum*, *Trichoderma*, *Paenibacillus*) enhanced fruit set, yield and quality in apple. These findings collectively substantiate the yield benefits observed in the current study under integrated nutrient treatments.

### Soil characteristics

The results indicated that soil pH remained statistically non-significant across the treatments, with values ranging from 6.77 in T_6_ (RDF (75%) + UHF Jeevanu Khad (25%)) to 7.11 in T_2_ (vermicompost (40%) + poultry manure (60%)). Electrical conductivity (EC) also exhibited no significant differences among treatments (Table 1). The lowest EC value of 0.23 dS m^− 1^ was observed in T_1_ [vermicompost (50%) + poultry manure (50%)], whereas the highest (0.27 dS m^− 1^) was recorded in T_6_ and T_7_. In contrast, soil organic carbon content was significantly influenced by the applied nutrient combinations. T_3_ (vermicompost (60%) + poultry manure (40%)) resulted in the highest organic carbon level (1.23%), while the lowest (1.06%) was found in T_7_, indicating a clear advantage of organic inputs over chemical fertilization alone.

**Table 1:** Effect of different organic and integrated nutrient treatments on soil properties in kiwifruit cv. Allison

Treatment	pH	EC	OC (%)	*N* (kg ha^− 1^)	*P* (kg ha^− 1^)	K (kg ha^− 1^)	Fe (ppm)	Zn (ppm)	Cu (ppm)	Mn (ppm)
T_1_	6.89^ab^	0.23^ab^	1.21^a^	312.00^ab^	94.53^ab^	324.17^d^	60.17^ab^	3.81^b^	3.49^a^b	15.07^b^
T_2_	7.11^a^	0.24^ab^	1.20^a^	310.05^ab^	91.45^ab^	316.33^de^	57.41^abc^	3.63^c^	3.38^b^	14.43^c^
T_3_	6.97^ab^	0.25^ab^	1.23^a^	318.45^ab^	97.79^ab^	340.00^c^	62.78^a^	4.03^a^	3.76^a^	15.66^a^
T_4_	6.86^ab^	0.24^ab^	1.18^a^	327.20^ab^	103.22^ab^	355.83^ab^	55.08^abc^	3.49^cd^	3.34^b^	13.82^d^
T_5_	6.84^ab^	0.26^ab^	1.15^a^	290.45^b^	88.50^b^	303.50^e^	51.28^bcd^	3.36^de^	3.07^c^	13.01^e^
T_6_	6.77^b^	0.27^a^	1.11^a^	331.55^a^	107.29^a^	365.83^a^	49.59^cd^	3.25^ef^	3.01^c^	12.09^f^
T_7_	6.82^ab^	0.27^a^	1.06^a^	322.20^ab^	100.87^ab^	342.50^bc^	45.92^d^	3.09^f^	2.84^c^	10.65^g^

T_1_, Vermicompost (50%) + Poultry manure (50%); T_2_, Vermicompost (40%) + Poultry manure (60%); T_3_, Vermicompost (60%) + Poultry manure (40%); T_4_, *Jeevamrit* @ 30 L per vine + *Ghan jeevamrit* @ 3 kg per vine; T_5_, UHF- Jeevanu Khad @ 5 L per vine; T_6_, RDF (75%) + UHF- Jeevanu Khad (25%); T_7_, RDF (800:600:800 g vine^− 1^)

Values sharing the same letter within a column are not significantly different according to Duncan’s Multiple Range Test (*p* ≤ 0.05)

Available nitrogen content was highest in T_6_ (331.55 kg ha^− 1^), indicating the enhanced nitrogen status under integrated application of 75% RDF + UHF-Jeevanu Khad. Treatments such as T_4_ (*Jeevamrit* + *Ghan Jeevamrit*) and T_7_ (100% RDF) also showed comparatively high nitrogen levels, while T_5_ (UHF Jeevanu Khad @ 5 L vine^− 1^) recorded the lowest value (290.45 kg ha^− 1^), suggesting that sole biofertilizer application was insufficient for optimal nitrogen buildup. A similar response was observed for phosphorus and potassium (Table 1), where T_6_ recorded the highest P (107.29 kg ha^− 1^) and K (365.83 kg ha^− 1^), in contrast to the lowest levels found under T_5_. In the case of micronutrients, T_3_ (Vermicompost 60% + Poultry manure 40%) outperformed all treatments, showing the highest soil levels of Fe (62.78 ppm), Zn (4.03 ppm), Cu (3.76 ppm) and Mn (15.66 ppm).

The results showed that soil pH and EC were not significantly influenced by the tested treatments. Similar nonsignificant effects of organic and biofertilizer inputs on soil pH and EC have been reported earlier [56, 57]. However, a marginal decrease in pH was observed in T_6_ (75% RDF + UHF-Jeevanu Khad), which can be attributed to the release of organic acids during microbial decomposition, a common feature under integrated nutrient management systems [58–60].

Significant improvements were recorded in soil organic carbon under T_3_ (vermicompost 60% + poultry manure 40%), indicating the positive contribution of organic amendments to carbon buildup and soil organic matter restoration. This is supported by previous studies highlighting the role of vermicompost and FYM in enhancing soil organic carbon pools through microbial biomass enrichment and humification processes [61–64].

Macronutrient availability (N, P, K) was highest in T_6_, highlighting the synergistic role of *Bacillus*-based biofertilizers in promoting nitrogen fixation, phosphorus solubilization and potassium mobilization [65–68]. These mechanisms likely accelerated nutrient mineralization and improved nutrient availability even with a reduced proportion of chemical fertilizers. In contrast, the low macronutrient levels recorded under T_5_ (biofertilizer alone) indicate that while *Bacillus*-driven nutrient mobilization is beneficial, it may not sufficiently meet the nutritional demands of a high-biomass plantation crop like kiwifruit without supplemental nutrient sources.

Although direct field evidence for *Bacillus*-based consortia enhancing kiwifruit yield is limited, several studies in kiwifruit and related fruit crops have shown improved soil nutrient dynamics, stress tolerance, and plant health under Bacillus inoculation, traits closely linked with improved productivity [69, 70]. These findings further support the observed effectiveness of integrated nutrient management strategies such as T_6_ in sustaining soil fertility and meeting crop nutrient requirements.

Micronutrient enrichment (Fe, Zn, Cu, Mn) under T_3_ highlights the chelating action of humic and fulvic acids released through organic manures [71, 72]. These compounds enhance micronutrient solubility and uptake, a trend consistent with previous findings in orchard ecosystems [73–76].

These results collectively suggest that integrated nutrient management, especially combinations of chemical fertilizers, organic inputs and beneficial microbes, can improve soil chemical properties and nutrient availability more effectively than individual components, reinforcing their relevance for sustainable fruit production.

### Microbial communities

**Fig. 4:**
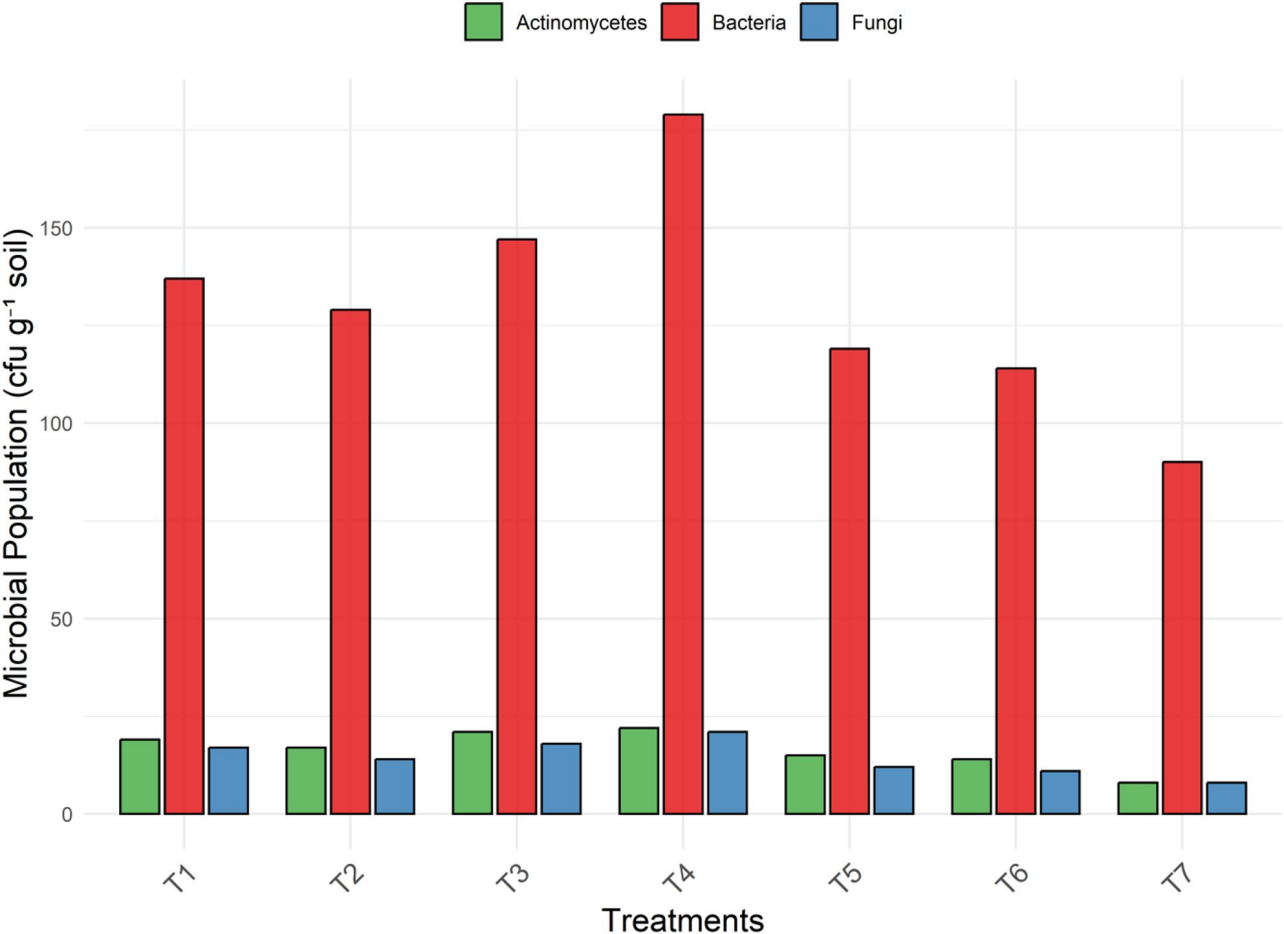
Effect of different nutrient treatments on soil microbial populations. Note: Bacteria, fungi, and actinomycetes were enumerated at dilution factors of 10⁵, 10³, and 10⁴ respectively; values represent treatment-wise trends and are not plotted on a uniform dilution basis. *T₁, Vermicompost (50%) + Poultry manure (50%); T₂, Vermicompost (40%) + Poultry manure (60%); T₃, Vermicompost (60%) + Poultry manure (40%); T₄, Jeevamrit @ 30 L per vine + Ghan jeevamrit @ 3 kg per vine; T₅, UHF- Jeevanu Khad @ 5 L per vine; T₆, RDF (75%) + UHF- Jeevanu Khad (25%); T₇, RDF (800:600:800 g vine⁻¹)*

The microbial population in the soil exhibited considerable variation across treatments, reflecting the influence of different nutrient combinations on soil biological activity (Fig. 4). Bacterial counts ranged from 90 to 179 × 10^5^ cfu g^− 1^ soil, with higher populations generally observed in treatments that included organic inputs. Treatments such as T_1_, T_3_ and T_4_ supported enhanced bacterial activity, while the lowest population was noted in T_7_, which received only chemical fertilizers. Fungal populations also varied significantly, with counts ranging between 10 and 23 × 10^3^ cfu g^− 1^. Treatments like T_3_ and T_4_ recorded relatively higher fungal populations, whereas T_6_ and T_7_ showed reduced counts, indicating limited fungal proliferation under predominantly inorganic regimes. Actinomycetes populations followed a similar trend, showing a range from 8 to 22 × 10^4^ cfu g^− 1^. Moderate counts were observed in T_1_ and T_2_, while higher populations were noted in T_4_. The lowest actinomycetes count was recorded in T_7_.

**Fig. 5:**
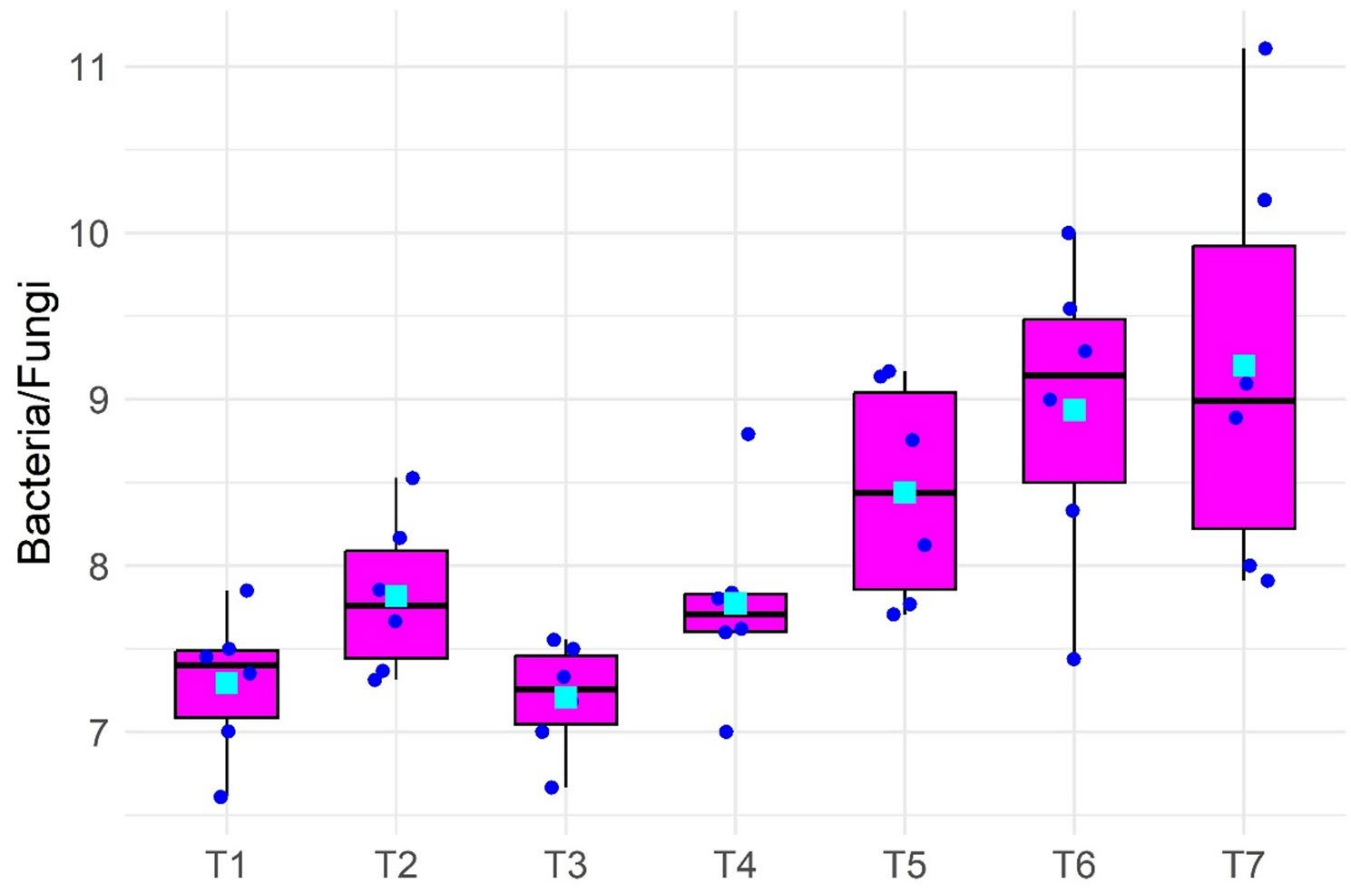
Boxplot illustrating the Bacteria/Fungi ratio under different nutrient management treatments. Medians (black lines), means (cyan squares) and individual points (blue dots). *T₁, Vermicompost (50%) + Poultry manure (50%); T₂, Vermicompost (40%) + Poultry manure (60%); T₃, Vermicompost (60%) + Poultry manure (40%); T₄, Jeevamrit @ 30 L per vine + Ghan jeevamrit @ 3 kg per vine; T₅, UHF- Jeevanu Khad @ 5 L per vine; T₆, RDF (75%) + UHF- Jeevanu Khad (25%); T₇, RDF (800:600:800 g vine⁻¹)*

The ratio of bacterial to fungal populations in soil varied distinctly across treatments, as shown in Fig. 5. Lower Bacteria/Fungi ratios were observed in T_1_, T_2_, T_3_ and T_4_, with median values generally ranging between 7.2 and 7.8. T_3_ showed the most stable ratio with minimal variation. In contrast, higher ratios were noted in T_5_, T_6_ and T_7_, with T_7_ recording the highest variability and median ratio approaching 9.0. The bacteria-to-fungi ratio reflects soil microbial balance and nutrient dynamics. Lower ratios in T_1_-T_4_ indicate fungal dominance, typically associated with organic amendments that enhance carbon sequestration and soil structure, beneficial for perennial crops like kiwifruit. In contrast, higher ratios in T_5_-T_7_, especially under sole chemical fertilization, suggest bacterial dominance and faster but less sustainable nutrient cycling.

The use of *Jeevamrit* and *Ghan Jeevamrit* provides a biologically intensive and low-cost approach for improving soil health through microbial enrichment. These inputs supply diverse populations of beneficial microbes, including phosphate-solubilizing, nitrogen-fixing and plant growth-promoting microorganisms, which collectively enhance nutrient availability and support crop growth.

Recent microbial profiling studies have confirmed that *Jeevamrit* harbors a complex and functionally active microbiome dominated by *Bacillus*, *Pseudomonas*, *Azotobacter* and Actinomycetes, contributing to nutrient cycling and plant growth regulation. It was demonstrated that *Jeevamrit* contains multiple functional microbial guilds involved in nitrogen fixation, cellulose degradation, and phosphate solubilization, offering a sustained supply of metabolically active microbes to the rhizosphere [17]. Similarly, *Bacillus* and *Pseudomonas* were the key functional genera in *Jeevamrit*, contributing to IAA production, siderophore release and organic acid-mediated nutrient solubilization under zero-budget natural farming systems [18].

These mechanistic observations align with the present study, where *Jeevamrit*-based treatments (T_4_) resulted in higher microbial counts and improved soil biological activity. Increased bacterial and fungal populations under *Jeevamrit* application have earlier been associated with enhanced soil organic carbon, which serves as a substrate for microbial proliferation [16]. Integrating *Jeevamrit* with organic nutrient sources significantly improved soil quality index, microbial biomass carbon, and system productivity in a rice-mustard-green gram rotation, reinforcing its role as a biologically regenerative input [77].

Multiple studies support the microbial enhancement effect observed here: increased nitrogen-fixing microbial populations [28], stimulation of soil respiration and biological activity under natural input regimes [78] and documented rises in enzyme activity, nutrient mineralization and microbial diversity following *Jeevamrit* application [79–82]. Complementary effects of vermicompost and microbial inoculants on microbial biomass and nutrient absorption [83], as well as improved soil structural and enzymatic stability under vermicompost amendment [84, 85], further validate the synergistic advantage of organic biostimulants such as those used in T_4_.

### Correlation studies

**Fig. 6:**
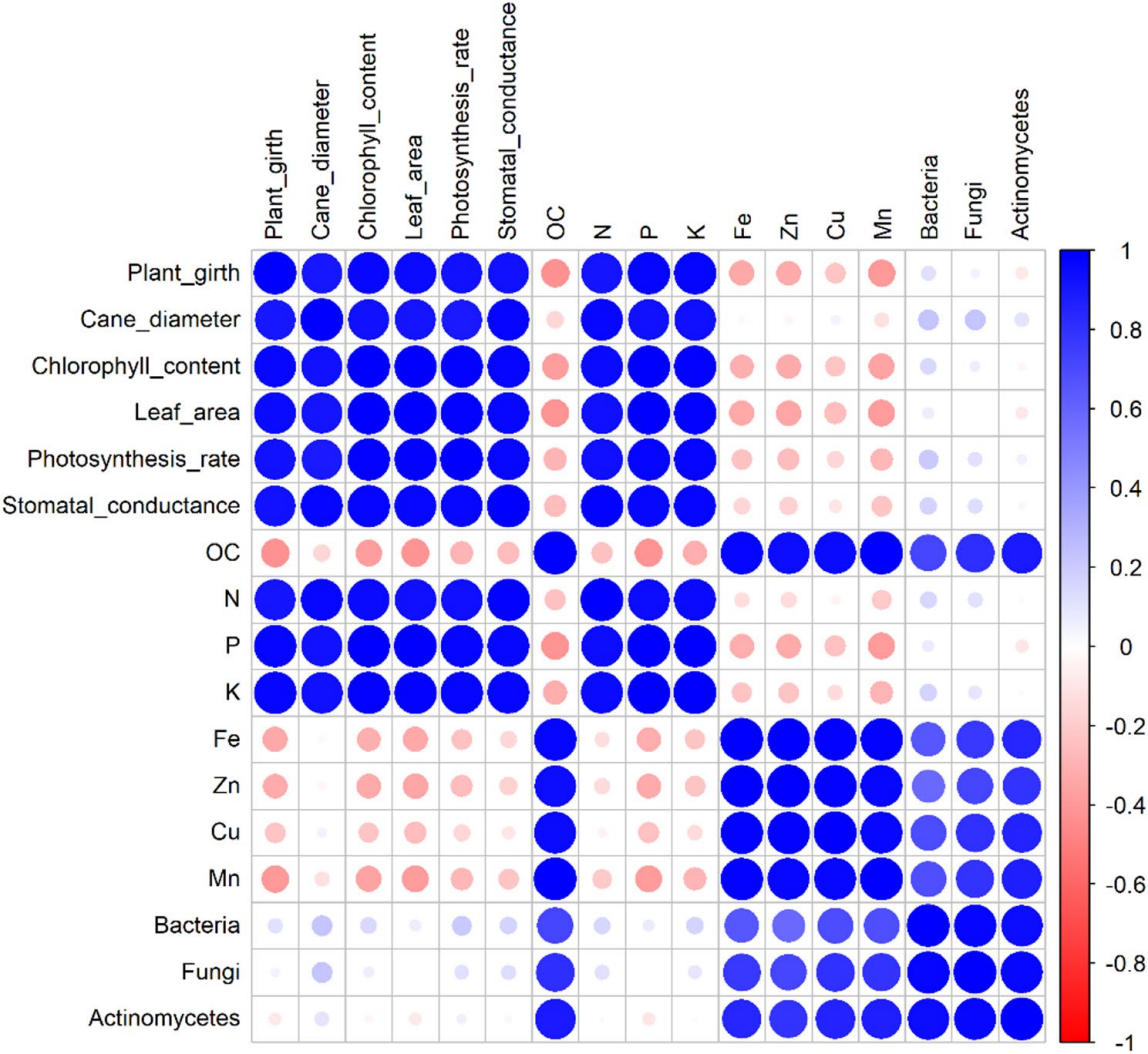
Heatmap showing correlations among plant traits, soil nutrients and microbial properties. Circle size and color indicate correlation strength and direction, blue for positive, red for negative.

A clear pattern of positive associations was observed among the vegetative and physiological parameters of the plant (Fig. 6). Plant girth exhibited strong correlations with cane diameter (*r* = 0.97), chlorophyll content (*r* = 0.96), leaf area (*r* = 0.93) and photosynthetic rate (*r* = 0.93), reflecting a consistent linkage among growth traits. Chlorophyll content was highly correlated with both leaf area (*r* = 0.99) and photosynthetic rate (*r* = 0.98), while leaf area also showed a robust correlation with cane diameter (*r* = 0.99), indicating close alignment among foliar development and assimilatory processes. In terms of nutrient relationships, available N, P and K were tightly associated. Available N correlated strongly with available P (*r* = 0.99) and K (*r* = 0.99), while available P also showed a strong association with available K (*r* = 0.99), highlighting balanced macronutrient dynamics in the system. Soil pH showed a negative correlation with available N (*r* = -0.38), P (*r* = -0.43) and K (*r* = -0.47), suggesting that mild acidification under organic and integrated treatments promoted nutrient solubilization and mobilization. Soil OC demonstrated strong positive correlations with several micronutrients, including Fe (*r* = 0.97), Zn (*r* = 0.95), Cu (*r* = 0.95) and Mn (*r* = 0.95), suggesting a collective enhancement in micronutrient status alongside increased organic matter content. Biological indicators of soil fertility were also significantly interlinked with nutrient parameters. Bacterial populations were positively correlated with OC (*r* = 0.72), Fe (*r* = 0.69) and Zn (*r* = 0.71). Fungal abundance showed strong correlations with OC (*r* = 0.81) and Mn (*r* = 0.82), whereas actinomycetes exhibited similar strong positive correlations with OC (*r* = 0.81), Fe (*r* = 0.80) and Zn (*r* = 0.80).

Conversely, certain negative correlations were observed, particularly between some micronutrients and growth-related parameters. Fe showed negative correlations with plant girth (*r* = -0.33), cane diameter (*r* = -0.30), chlorophyll (*r* = -0.30) and leaf area (*r* = -0.33). Simultaneously, Zn also showed negative associations with plant girth (*r* = -0.32) and chlorophyll (*r* = -0.32), while copper was negatively correlated with leaf area (*r* = -0.36) and chlorophyll (*r* = -0.22). In addition, soil OC showed modest negative correlations with plant girth (*r* = -0.42), leaf area (*r* = -0.29) and chlorophyll (*r* = -0.41).

The observed correlations in the present study align with earlier findings. Kakar et al. [86] reported a negative correlation between soil K and leaf Ca, indicating nutrient antagonism. The inverse correlation between soil pH and available macronutrients confirms that slight reductions in pH favor the release of N, P, and K, likely through organic acid production and chelation mediated by phosphate and potassium-solubilizing *Bacillus* spp. This mechanism is well supported in biological soil management systems. Sharma and Verma [87] found positive associations of soil organic carbon with leaf Mn and Mg, highlighting the role of organic matter in micronutrient uptake. These results are in agreement with Dhiman et al. [88], who observed significant positive correlations between soil organic carbon, microbial abundance and nutrient availability across mango orchards in the Himalayan foothills. Reduced Fe availability under higher pH, as noted in this study, corresponds with findings by [89]. Strong correlations between leaf K, Zn, P, Fe, and Cu with fruit yield reported by Mehta et al. [1] further confirm the critical role of these nutrients. Mehta et al. [90] also linked post-harvest soil OC and available N, P, K with yield, emphasizing the contribution of actinobacteria and *Azotobacter* to nutrient dynamics.

### Multiple linear regression

A multiple linear regression model was fitted to predict yield using five explanatory variables: soil available N, chlorophyll content, photosynthetic rate, bacterial population and soil available K. The model yielded a high coefficient of determination (R^2^ = 0.9938) and an adjusted R^2^ of 0.9625; however, the overall model was not statistically significant (*p* = 0.1338), likely due to the limited sample size (*n* = 7).

**Fig. 7:**
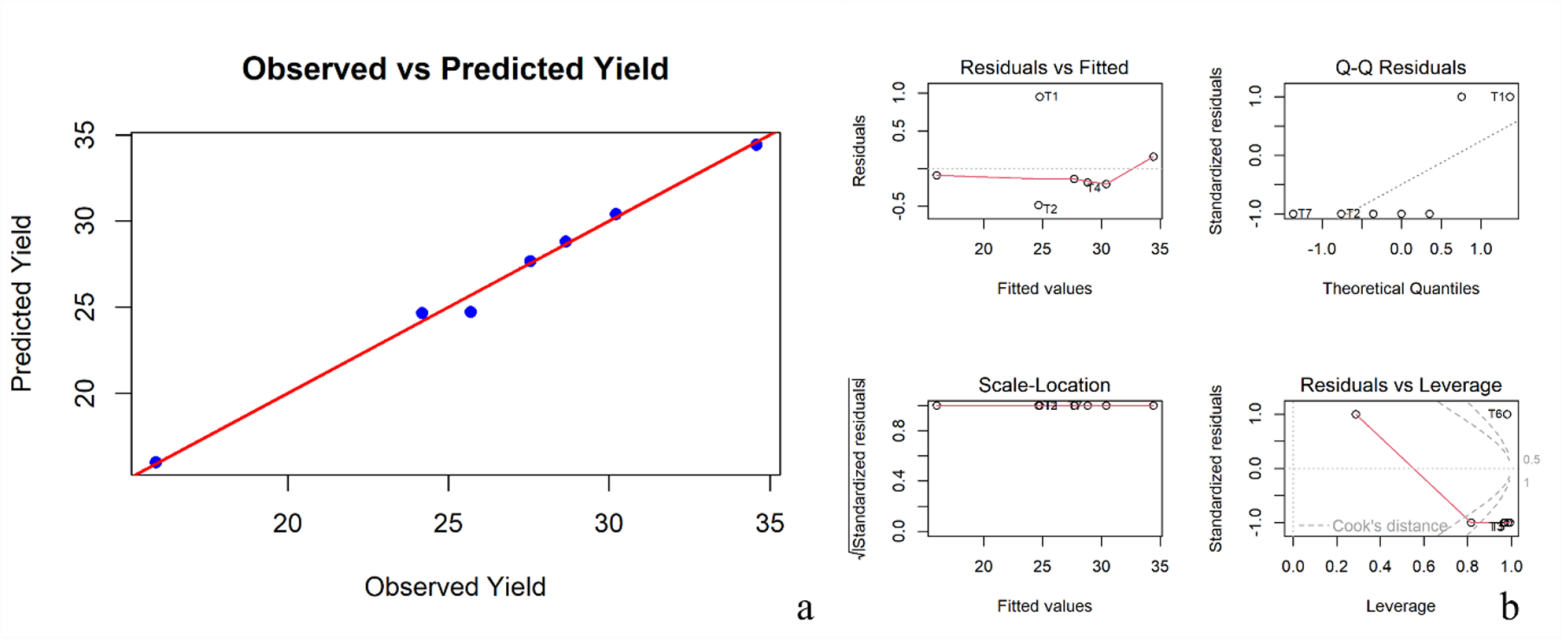
Observed vs. predicted yield (**a**) and diagnostic plots (**b**) from multiple regression analysis

The observed versus predicted yield plot displayed a close alignment (slope near 1), indicating correspondence between observed and fitted values within the dataset. Diagnostic plots further confirmed no major violations of regression assumptions, with residuals exhibiting normal distribution, constant variance and no influential data points (Fig. 7a and b). The F-statistic for the overall model was 31.82 (*p* = 0.1338), reflecting limited statistical significance under the small sample size (*n* = 7). Among the predictors, available N emerged as the most influential variable with a positive regression coefficient (0.441) and the highest absolute t-value (3.615), indicating a strong linear relationship with yield.

Photosynthetic rate (t = 1.306) and chlorophyll content (t = -1.056) showed moderate influence, while bacterial population (t = -1.157) and available K (t = 0.037) contributed relatively little to yield prediction. The observed versus predicted yield plot displayed a near-perfect alignment (slope close to 1), indicating close agreement between observed and fitted values within the dataset. Model diagnostics revealed no major violations of regression assumptions. The residuals were symmetrically distributed and exhibited constant variance across fitted values. The Q-Q plot showed residuals following the normality assumption. Leverage and Cook’s distance plots indicated no significant outliers or influential observations.

Overall, the regression analysis should be interpreted as exploratory rather than predictive, indicating directional relationships between yield and the selected variables. Available N emerged as the most influential predictor, indicating its comparatively stronger association with yield, mirroring outcomes in maize models where N and chlorophyll content dominated yield prediction. This aligns with findings from fruit crop systems, where nitrogen status and physiological traits such as chlorophyll content and photosynthetic rate have been strongly associated with yield performance in apple [91], mango and citrus [92] and mixed orchard systems [93]. The relatively modest impact of bacterial population and available K suggests possible threshold effects or limited variance among treatments [94, 95]. Prior research has shown that microbial indicators may have secondary predictive power depending on context and microbial diversity level. These findings highlight the integrative value of combining physiological traits (e.g., chlorophyll, photosynthesis), soil nutrient status (especially N), and microbial indicators for robust yield modeling. They emphasize that strategies optimizing N availability and physiological function can substantially improve productivity, while microbial and K contributions may depend on environmental context and baseline nutrient levels.

### Heatmap clustering analysis

**Fig. 8:**
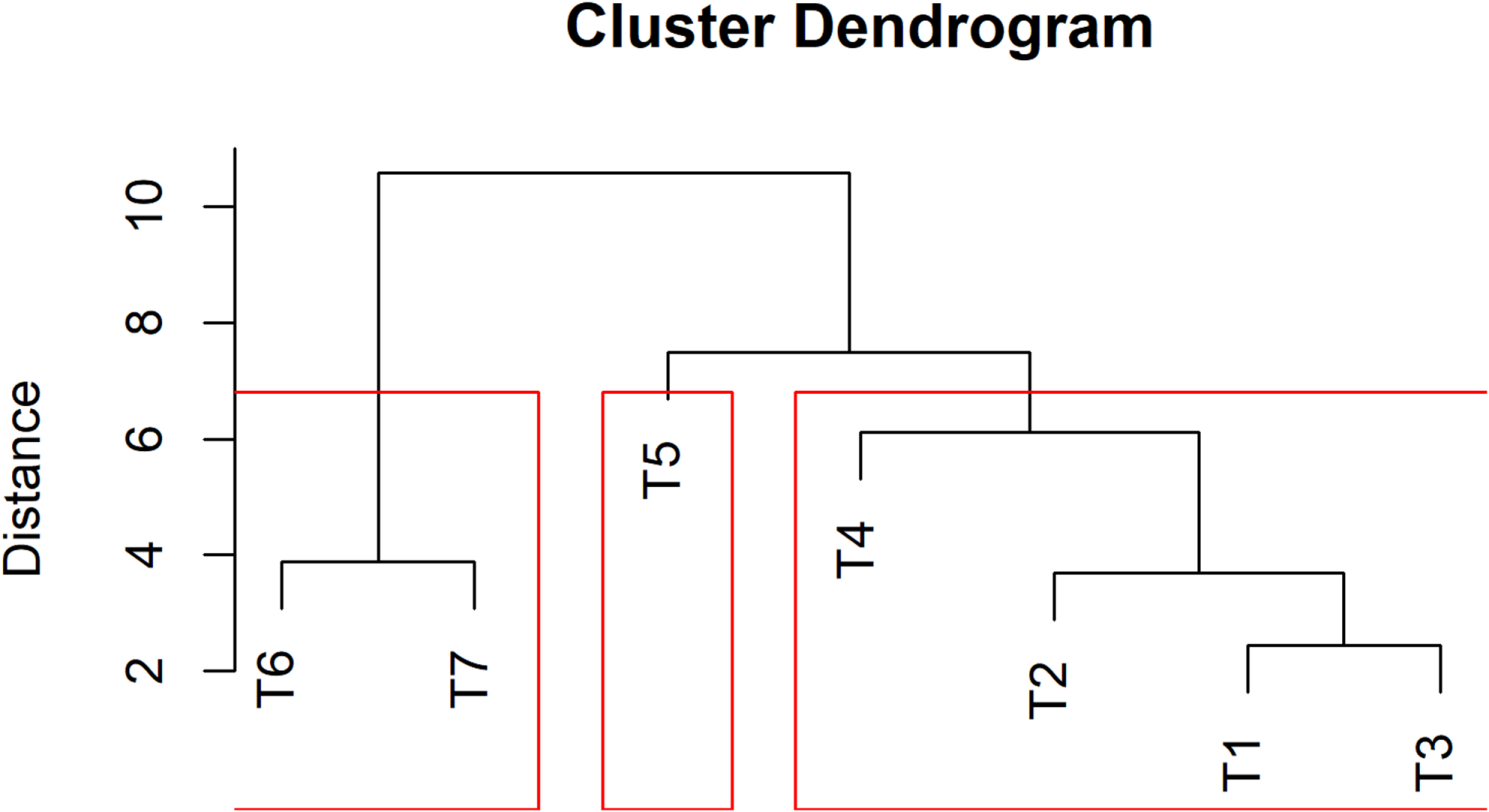
Cluster dendrogram of nutrient regimes based on soil, microbial and physiological traits. The red line indicates three major clusters. *T₁, Vermicompost (50%) + Poultry manure (50%); T₂, Vermicompost (40%) + Poultry manure (60%); T₃, Vermicompost (60%) + Poultry manure (40%); T₄, Jeevamrit @ 30 L per vine + Ghan jeevamrit @ 3 kg per vine; T₅, UHF- Jeevanu Khad @ 5 L per vine; T₆, RDF (75%) + UHF- Jeevanu Khad (25%); T₇, RDF (800:600:800 g vine⁻¹)*

Hierarchical cluster analysis grouped the seven treatments into three distinct clusters based on their soil, microbial, and physiological trait profiles (Fig. 8). Cluster I included T_1_, T_2_, T_3_, and T_4_ which exhibited generally moderate to low levels of microbial populations, nutrient concentrations, and physiological parameters. Among them, T_3_ showed the lowest overall performance, whereas T_1_ and T_2_ were closely associated, indicating similarity in their response. Cluster II comprised only T_5_, which formed a separate group due to its distinct profile characterized by higher levels of electrical conductivity, Fe, Zn, and cane diameter, but relatively lower values for photosynthetic efficiency and leaf traits.

**Fig. 9:**
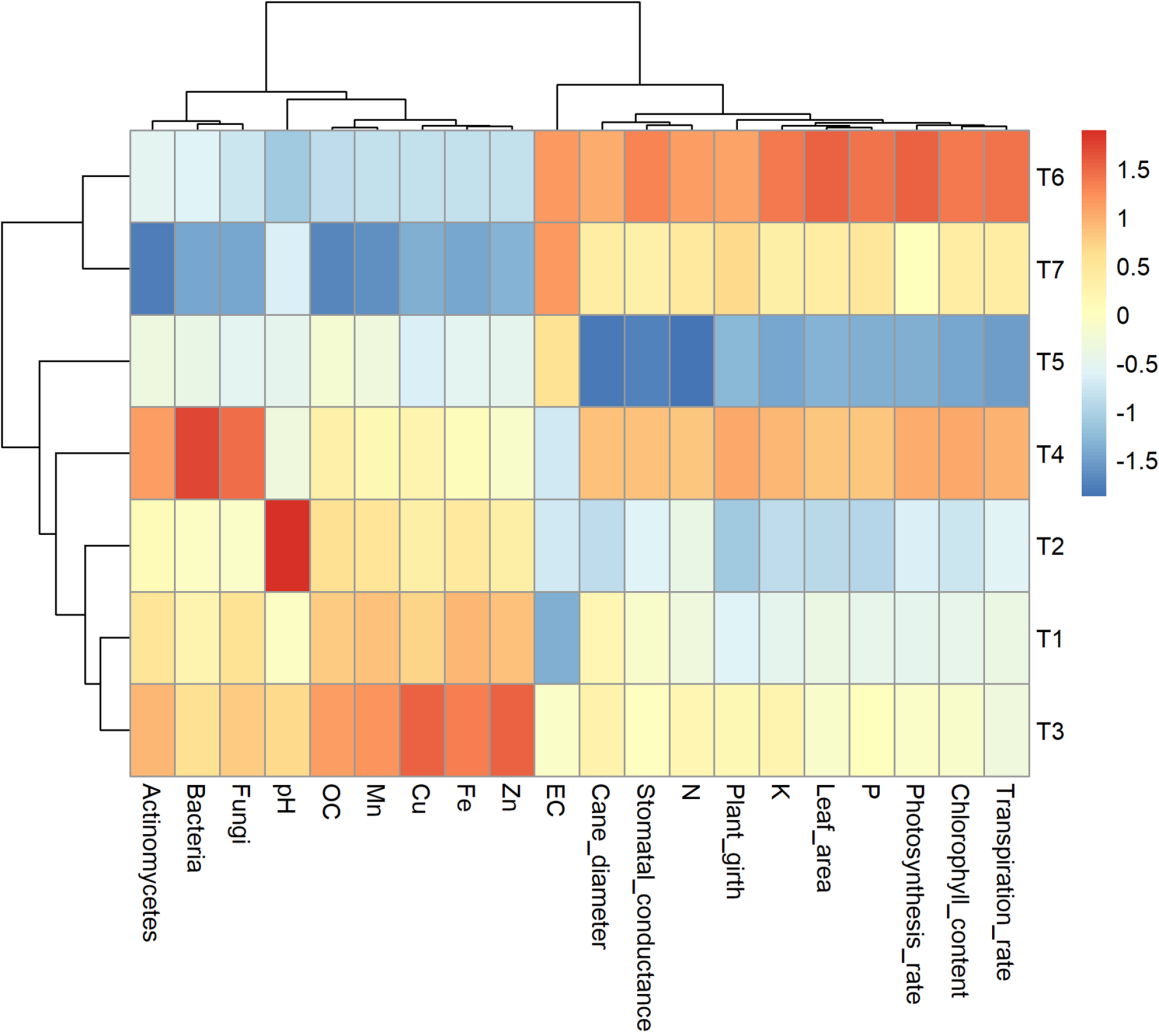
Heatmap showing hierarchical clustering of treatments and traits. Color intensity indicates the standardized values of each trait, with red for high and blue for low. *T₁, Vermicompost (50%) + Poultry manure (50%); T₂, Vermicompost (40%) + Poultry manure (60%); T₃, Vermicompost (60%) + Poultry manure (40%); T₄, Jeevamrit @ 30 L per vine + Ghan jeevamrit @ 3 kg per vine; T₅, UHF- Jeevanu Khad @ 5 L per vine; T₆, RDF (75%) + UHF- Jeevanu Khad (25%); T₇, RDF (800:600:800 g vine⁻¹)*

Cluster III consisted of T_6_ and T_7_, both of which demonstrated superior performance, with the highest standardized values for microbial populations (bacteria and fungi), chlorophyll content, photosynthetic rate, transpiration rate, stomatal conductance, biological activity and plant physiological functions. The heatmap with hierarchical clustering of both treatments and traits (Fig. 9) supported these groupings. Treatments T_6_ and T_7_ displayed intense red shades across most traits, indicating higher standardized values, while T_3_ and T_5_ showed predominance of blue shades, reflecting lower values. Trait clustering revealed co-associated parameters, where microbial counts and photosynthetic traits grouped together. The heatmap visually confirmed the differentiation among treatments based on the measured variables.

The hierarchical clustering and heatmap analyses effectively captured distinct treatment responses by integrating microbial, physiological, and soil trait data. Treatments T_6_ and T_7_ clustered together, characterized by high canopy physiological activity (chlorophyll, photosynthesis, transpiration) and elevated microbial populations, suggesting the synergistic effect of combining biofertilizers like UHF-Jeevanu Khad with partial chemical fertilizer applications. In contrast, treatments grouped in Clusters I and II reflected poorer physiological and microbial performance, associated with lower soil fertility or nutrient imbalances. This pattern aligns with findings from Paramesh et al. [75], where clustering of integrated nutrient management treatments revealed co-regulated enhancements in physiological efficiency and microbial biomass. Additionally, Garcia-Perez et al. [96] demonstrated that labels treated with vermicompost biostimulants exhibited coherent clustering of biochemical traits and physiological upregulation, further validating the use of fold-change heatmaps to reveal treatment synergy across microbial and plant traits.

### Principal component analysis

**Fig. 10:**
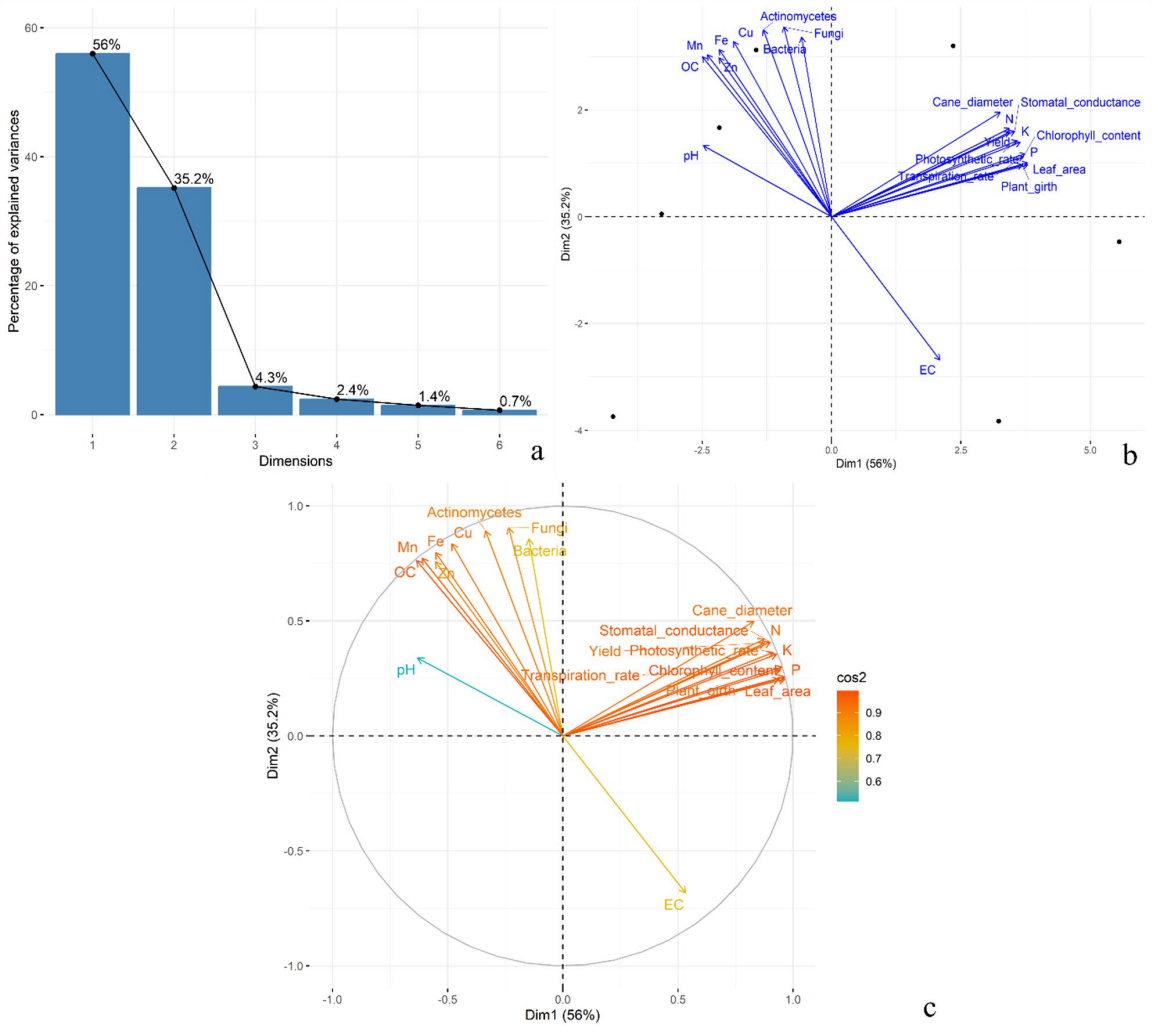
PCA scree plot (**a**), biplot (**b**) and correlation circle (**c**) showing variance explained, treatment distribution and trait contributions

Principal Component Analysis (PCA) was performed to assess the multivariate relationships among soil, microbial and physiological traits. The scree plot (Fig. 10a) indicated that the first two principal components, PC1 and PC2, accounted for 55.98 and 35.17 per cent of the total variation, respectively, cumulatively explaining 91.15 per cent of the dataset variance. PC3 and PC4 contributed marginally, with 4.35 and 2.39 percent variance, respectively (Table 2). The PCA biplot (Fig. 10b) revealed clear separation of treatments along the first two dimensions. Treatments T_6_ and T_7_ were positioned positively along PC1 and PC2, closely aligned with traits such as photosynthetic rate, chlorophyll content, transpiration rate, leaf area and nutrient contents (available N, P, K), indicating their association with improved physiological and nutritional performance. On the other hand, T_5_ was located on the negative side of PC2, aligning more with EC and less with physiological attributes. T_3_ and T_2_ showed relative association with micronutrients (Fe, Mn, Cu) and organic carbon. The correlation circle (Fig. 10c) further illustrated the strength and direction of relationships among variables. Variables located near the perimeter of the circle, such as leaf area, available P, chlorophyll content and transpiration rate, had high cos^2^ values, indicating strong representation on the PC1-PC2 plane. Traits such as fungi (0.905), actinomycetes (0.891), bacteria (0.857) and micronutrients (Cu, Fe, Mn) clustered together along PC2, suggesting a microbial-nutrient interaction axis. In contrast, EC showed a strong negative loading on PC2 (-0.683), indicating its inverse relationship with microbial and physiological traits.

**Table 2:** Eigenvalues, explained variance and factor loadings of soil, microbial and physiological traits on PC1 to PC4

	Principal component			
	PC1	PC2	PC3	PC4
Eigen Value	11.76	7.39	0.91	0.5
Variability (%)	55.98	35.17	4.35	2.39
Cumulative Variance (%)	55.98	91.15	95.5	97.89
Yield contributing traits	Factor loadings			
	F1	F2	F3	F4
Plant girth	0.94	0.247	-0.081	0.084
Cane diameter	0.829	0.498	0.103	0.056
Chlorophyll content	0.951	0.299	-0.038	-0.048
Leaf_area	0.964	0.247	-0.03	0.051
Photosynthetic rate	0.913	0.363	-0.039	-0.037
Stomatal conductance	0.901	0.408	0.106	-0.052
Transpiration rate	0.944	0.287	0.015	-0.14
pH	-0.631	0.339	0.508	-0.407
EC	0.533	-0.683	0.067	0.34
OC	-0.635	0.762	0.035	0.048
N	0.875	0.421	0.209	-0.088
P	0.964	0.257	0.002	0.067
K	0.928	0.355	0.012	0.07
Fe	-0.553	0.797	0.187	0.146
Zn	-0.554	0.758	0.229	0.256
Cu	-0.482	0.835	0.175	0.181
Mn	-0.612	0.773	0.084	0.116
Bacteria	-0.148	0.857	-0.445	-0.156
Fungi	-0.235	0.905	-0.335	-0.067
Actinomycetes	-0.336	0.891	-0.273	0.014
Yield	0.876	0.404	0.255	-0.034

*PC1* Principal component-1, *PC2* Principal component-2, *PC3* Principal component-3, *PC4* Principal component-4, *F1* factor-1, *F2* factor-2, *F3* factor-3, *F4* factor-4, *EC* electrical conductivity, *OC* organic carbon

Overall, the PCA revealed that PC1 captured variability primarily related to plant physiological and yield traits, while PC2 was defined by microbial counts and soil micronutrient content. The figures and tables collectively highlight the multidimensional influence of treatments across biological, chemical and physiological axes.

The PCA revealed that PC1 primarily captured variation associated with plant physiological and yield-related traits such as chlorophyll content, photosynthetic rate, transpiration rate and available NPK, indicating strong functional linkage with carbon assimilation and nutrient dynamics. This is consistent with findings by Kumar et al. [97], who observed that nutrient supply modes significantly influenced agronomic performance and trait expression in fruit crops. Similarly, Kumar et al. [98] reported that principal components effectively differentiated foliar nutrient responses and generative potential under variable nutrient regimes. The strong grouping of microbial populations (fungi, actinomycetes, bacteria) with micronutrients (Cu, Fe, Mn) along PC2 suggests a microbial, nutrient interaction axis, indicative of biological enhancement of nutrient cycling. These findings are aligned with Saini et al. [99], who found that rhizosphere microbial composition was closely associated with micronutrient availability and fruit quality. Moreover, Saini et al. [100] demonstrated that nano-nutrient interventions modulate plant physiological and antioxidant activity, contributing to trait clustering observed in PCA. The strong negative loading of EC on PC2 further underscores its antagonistic role in microbial and physiological trait expression, highlighting the suppressive effect of higher salinity levels on biological functioning.

## Conclusion

The present study demonstrates that integrated nutrient management practices combining organic manures, *Bacillus*-based biofertilizers and *Jeevamrit* formulations significantly enhance soil chemical properties, microbial activity and plant physiological performance in kiwifruit under mid-hill conditions. Among the treatments, T_6_ (75% RDF + UHF-Jeevanu Khad) and T_4_ (*Jeevamrit* + *Ghan Jeevamrit*) showed the most pronounced improvements in nutrient availability, chlorophyll content, photosynthetic efficiency, microbial populations and fruit yield, highlighting the efficacy of biologically enriched nutrient strategies. Multivariate analyses, including PCA and regression modelling, confirmed available nitrogen and photosynthetic traits as key determinants of yield, emphasizing the central role of harmonized nutrient and microbial management in orchard systems. The findings endorse the potential of organic and natural input-based modules to reduce dependence on synthetic fertilizers while improving ecosystem functioning.

Future research should aim to:

- Validate these nutrient modules in other fruit crops and diverse agro-ecological zones,
- Investigate long-term impacts on soil carbon sequestration and microbial diversity,
- Optimize microbial consortia tailored to specific orchard systems,
- Integrate these practices with climate-smart approaches for resilient, low-external-input horticulture.

## Supplementary Information

Supplementary Material 1.

## Data Availability

The datasets used and analyzed in the current study are available from the corresponding author on reasonable request.
