# Supplementary material for: Multivariate elucidation of soil-microbial-physiological interactions under bio-organic nutrient modules in kiwifruit (Actinidia deliciosa A. Chev.)
Source: BMC Plant Biol. 2026 Apr 2;26:651. doi: 10.1186/s12870-026-08259-6 (PMC13067591; doi:10.1186/s12870-026-08259-6)
Supplement: Supplementary file 1 — Supplementary Material 1. [file 12870_2026_8259_MOESM1_ESM.docx]

**Table S1 Composition of nutrients in organic fertilizers used in present study**

| **Organic fertilizers** | **N (%)** | **P (%)** | **K (%)** |
| --- | --- | --- | --- |
| Vermicompost | 1.14 | 0.71 | 0.58 |
| Poultry manure | 1.69 | 1.03 | 1.97 |
| *Jeevamrit* | 1.96 | 0.17 | 0.28 |
| *Ghan Jeevamrit* | 1.49 | 0.55 | 1.09 |

**Table S2 Coefficients of the multiple linear regression model predicting yield based on different parameters**

| **Predictor** | **Estimate** | **Standard Error** | **t Value** | **p-Value** |
| --- | --- | --- | --- | --- |
| Intercept | -87.7918 | 23.9231 | -3.670 | 0.169 |
| Available N | 0.4410 | 0.1220 | 3.615 | 0.172 |
| Chlorophyll Content | -18.3898 | 17.4105 | -1.056 | 0.483 |
| Photosynthesis Rate | 2.3964 | 1.8348 | 1.306 | 0.416 |
| Bacteria | -0.0207 | 0.0179 | -1.157 | 0.454 |
| Available K | 0.0056 | 0.1507 | 0.037 | 0.976 |


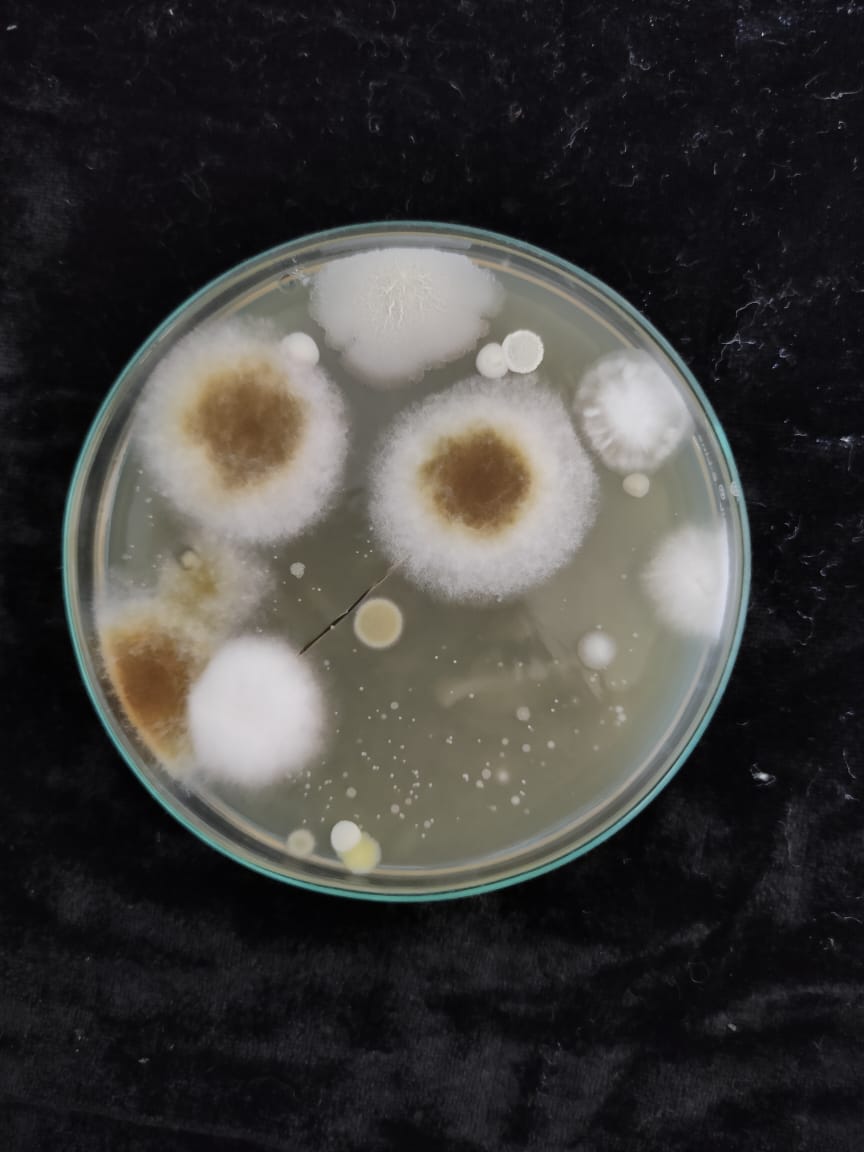

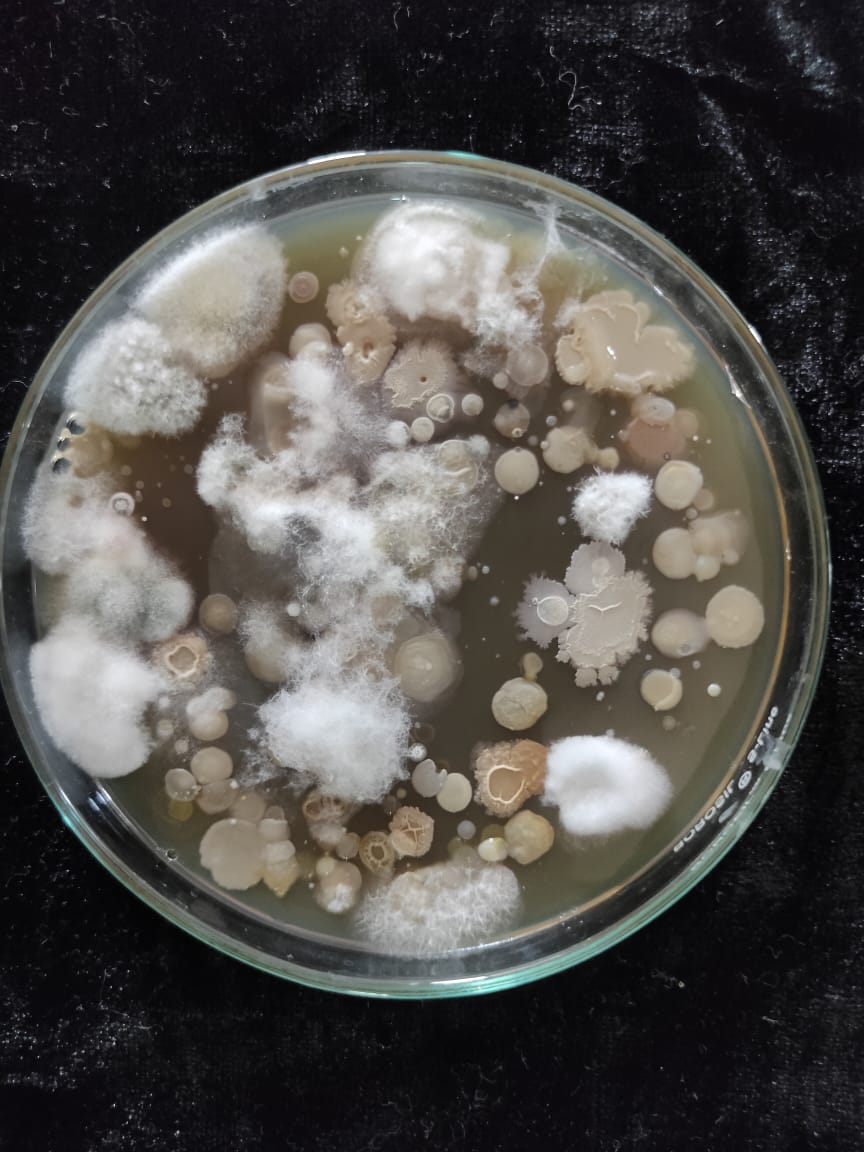


T_6_ (75% RDF + UHF-Jeevanu Khad) Fungi


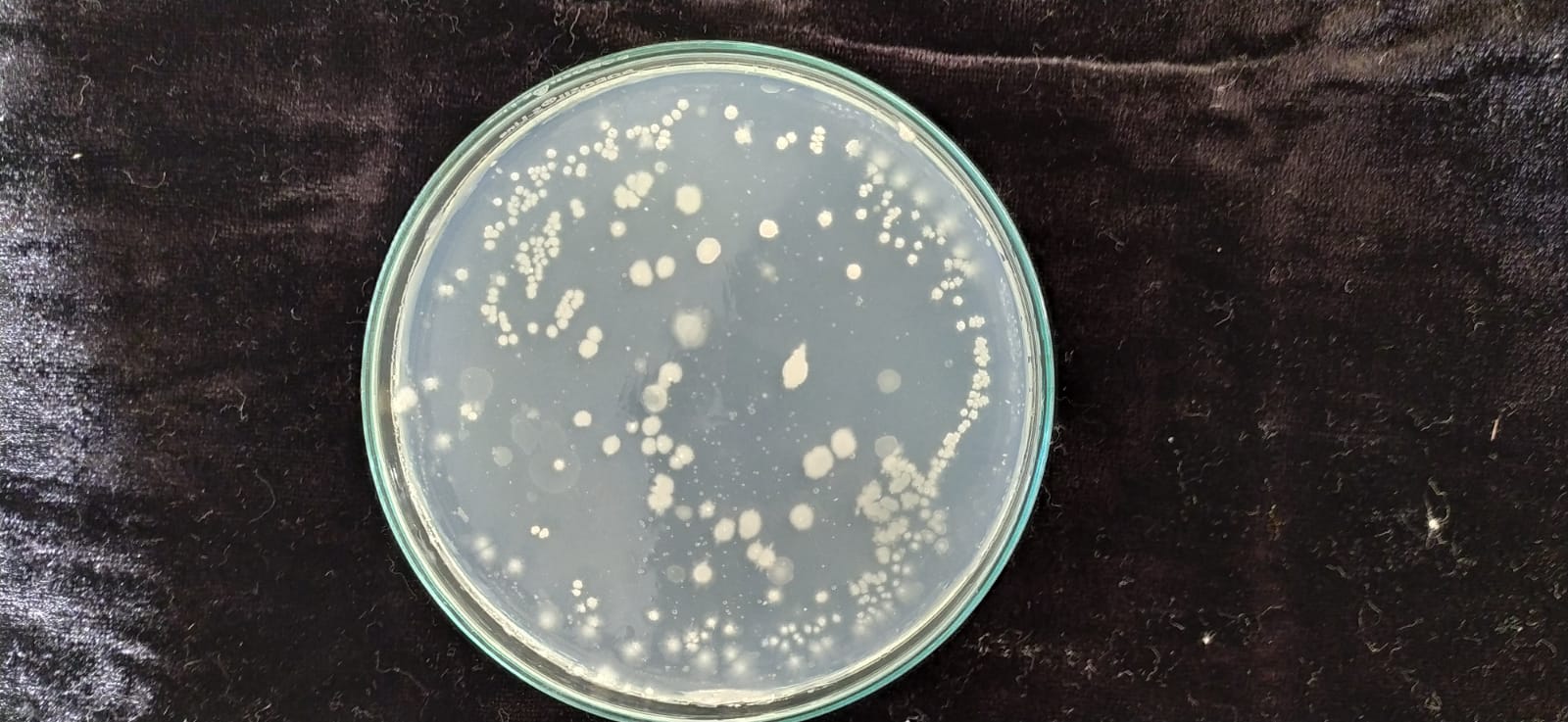

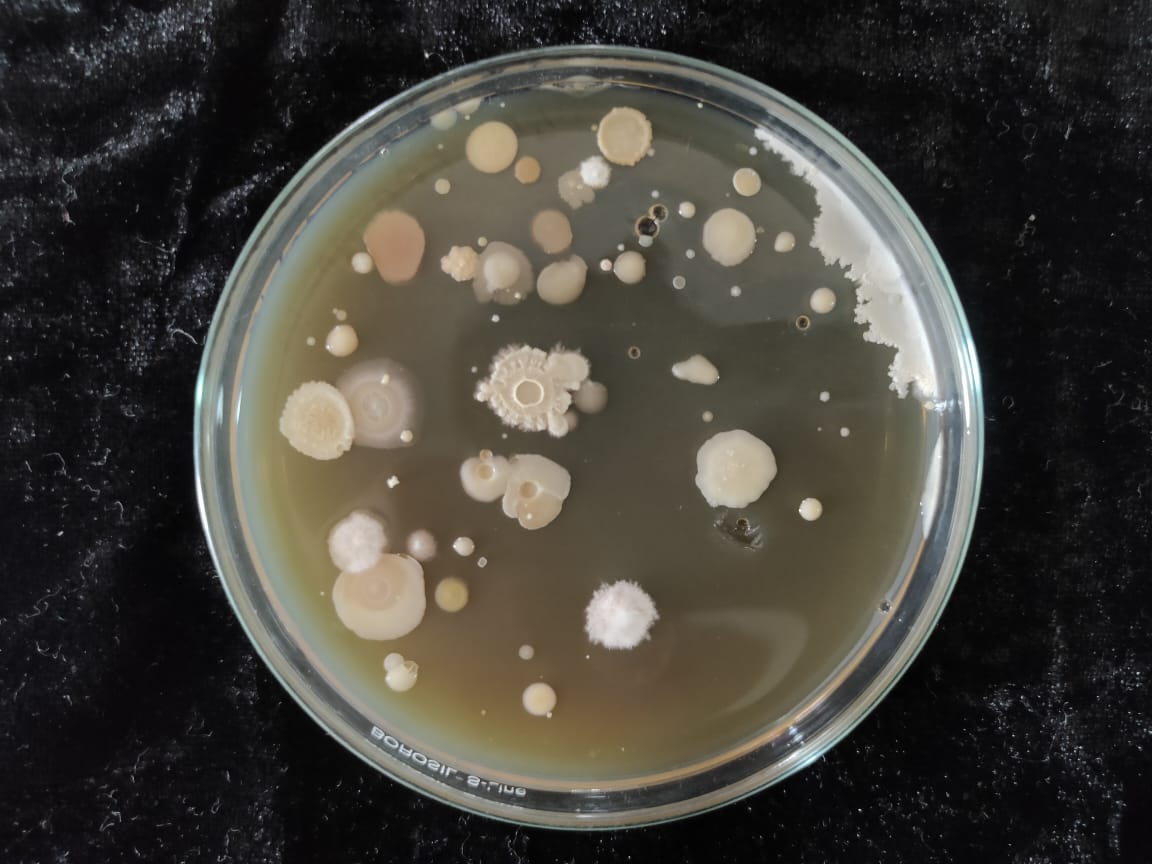


T_4_ (*Jeevamrit* + *Ghan Jeevamrit*) Bacteria T_2_ - Vermicompost (40%) + Poultry manure (60%) Bacteria


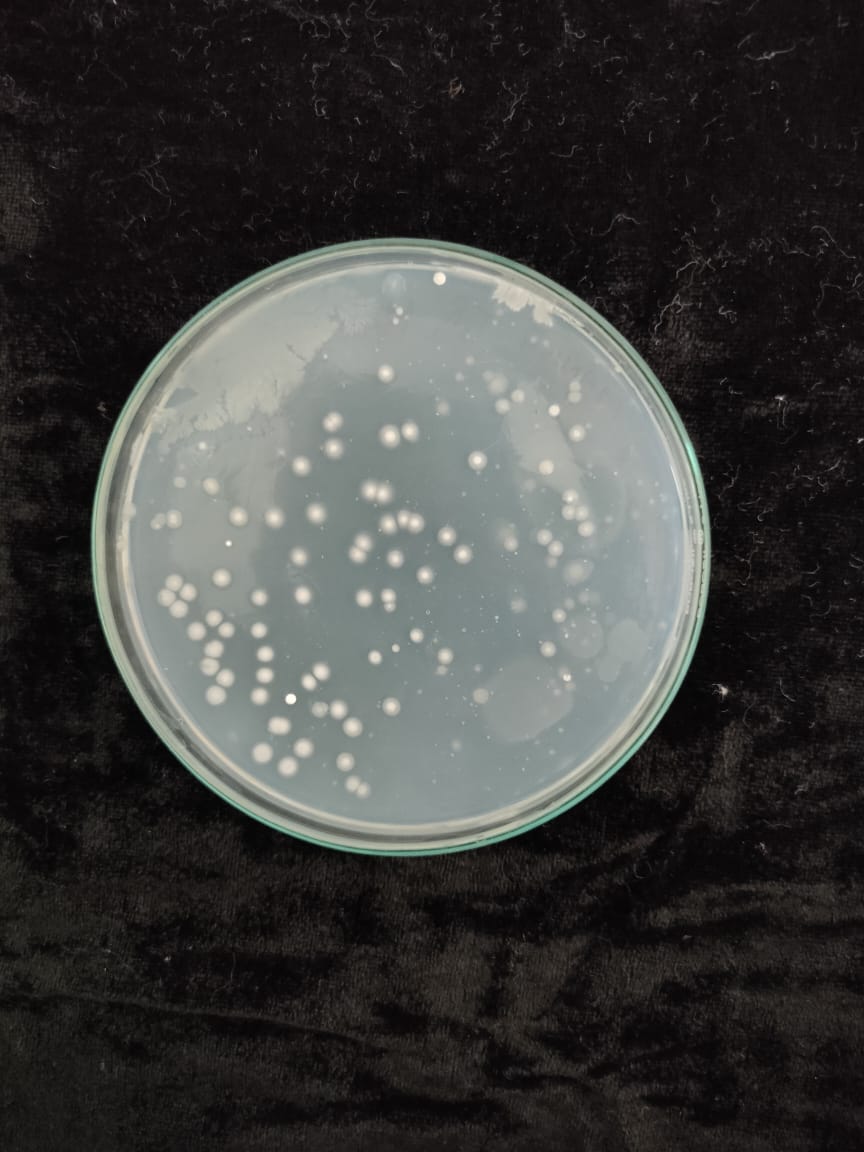


T_6_ (75% RDF + UHF-Jeevanu Khad) Actinomycetes

Figure 1. Plate showing different microbial colonies isolated from rhizosphere soil
